# Tracking localization and secretion of cellulase spatiotemporally and directly in living *Trichoderma reesei*

**DOI:** 10.1186/s13068-019-1538-0

**Published:** 2019-08-20

**Authors:** Chengcheng Li, Ai-Ping Pang, Hang Yang, Roujing Lv, Zhihua Zhou, Fu-Gen Wu, Fengming Lin

**Affiliations:** 1 State Key Laboratory of Bioelectronics, School of Biological Science and Medical Engineering, Southeast University, 37 Jinxianghe Road, Xuanwu District, Nanjing, 210096 Jiangsu China; 2 Key Laboratory of Synthetic Biology, Institute of Plant Physiology and Ecology, Shanghai Institutes for Biological Sciences, Chinese Academy of Sciences, Shanghai, 200032 China

**Keywords:** Protein secretion, Cellulolytic enzymes, Unconventional secretion pathway, Subcellular localization

## Abstract

**Background:**

Filamentous fungi secret hydrolytic enzymes like cellulase and hemicellulase outside the cells, serving as important scavengers of plant biomass in nature and workhorses in the enzyme industry. Unlike the extensive study on the mechanism of cellulase production in fungi, research on spatiotemporal distribution and secretion of cellulase in fungi is lacking, retarding the deeper understanding of the molecular mechanism behind the fungal cellulase production.

**Result:**

Recombinant *Trichoderma reesei* strains RBGL, RCBH, and RCMC were successfully constructed from *T. reesei* RUT-C30, expressing red fluorescent protein DsRed-tagged versions of β-glucosidase (BGL), cellobiohydrolase (CBH), and endoglucanase (CMC), respectively. With the assistance of these strains, we found that all three cellulase components BGL, CBH, and CMC diffused throughout the whole fungal mycelium with major accumulation at the hyphal apexes. These enzymes located in ER, Golgi, vacuoles and cell membrane/wall, but not septum, and secreted abundantly into the culture medium. Moreover, the major secretion of CBH and CMC started more early than that of BGL. Brefeldin A (BFA) completely blocked cellulase expression and secretion in *T. reesei.*

**Conclusion:**

Based on recombinant *T. reesei* RBGL, RCBH, and RCMC expressing DsRed-fused versions of BGL, CBH, and CMC, respectively, the distribution and secretion of cellulase production in *T. reesei* were first visualized directly in a dynamic way, preliminarily mapping the location and secretion of *T. reesei* cellulase and providing evidence for revealing the secretion pathways of cellulase in *T. reesei*. The obtained results suggest that cellulase excretion majorly occurs via the conventional ER–Golgi secretory pathway, and might be assisted through unconventional protein secretion pathways.

**Electronic supplementary material:**

The online version of this article (10.1186/s13068-019-1538-0) contains supplementary material, which is available to authorized users.

## Background

Filamentous fungi are evolved to possess efficient protein secretion and are widely used for industrial enzyme production [1]. For example, filamentous fungi like *Trichoderma*, *Penicillium,* and *Aspergillus* are extraordinary in their capability of cellulase/hemicellulase secretion for the efficient deconstruction of recalcitrant polymers in plant biomass including cellulose, hemicellulose, pectin, and lignin [2–4]. Nevertheless, the molecular mechanism remains unknown about the secretory pathways for extracellular enzymes in filamentous fungi. It would benefit the basic understanding of fungal cell biology and improvement of production strains if the sorting, targeting, and release of extracellular proteins of fungi are fully investigated.

Protein secretion in filamentous fungi can occur either by the conventional pathway traversing the endomembrane system or via other alternative routes termed as unconventional secretions [5]. Like other eukaryotic cells, the classical secretory pathway in filamentous fungi is the well-established endoplasmic reticulum (ER)–Golgi pathway. Proteins first enter the ER for folding, modification by glycosylation, disulfide bridge formation, phosphorylation, and subunit assembly before being packaged into transport vesicles for delivery to Golgi compartments. Once in Golgi, the proteins undergo further glycosylation by the addition and/or removal of specific sugar resides, and then are again packed in secretory vesicles and directed to the endosomal/vacuolar system, the Spitzenkörper, or the plasma membrane for exocytosis. In filamentous fungi, exocytosis majorly occurs at growing hyphal apexes where Golgi and ER compartments are accumulated abundantly [5]. Nevertheless, exocytosis at other sites of fungal cells, such as septa [6] and lateral plasma membrane [7, 8], has been observed. Besides the conventional pathway, the unconventional traffic pathways for protein secretion in fungi have also been reported, including Golgi-independent secretion which was BFA-insensitive [9], secretion independent of both ER and Golgi apparatus [10], and extracellular vesicle secretion [11]. For example, Stock et al. reported that the secretion of endochitinase in *Ustilago maydis* is through the unconventional secretion pathway, independent of the ER and the Golgi-like apparatus [10]. However, the mechanism for this unconventional pathway was not very clear.

*Trichoderma reesei* is of particular interest because of its remarkable cellulase secretion capability with the extracellular cellulase reaching up to 100 g/L [12]. Meanwhile, it is a model organism for studying the mechanism of cellulase production and secretion, as well as an attractive host for the production of heterologous proteins. Electron microscopic analysis of ER structure in *T. reesei RUT*-*C30* and QM6a is probably the earliest study on the protein secretory pathway of *T. reesei* [13]. Since then, efforts have been put on imaging and kinetics measurement of the secretion process [13–16], molecular characterization of the secretory pathway components [8, 17–22], and investigation of the relevant transcriptional regulation [23, 24]. The localization and secretion of cellulase has been studied by measurement of cellulase activity in subcellular fractionation [25], electron microscopy [13], and immunoelectron microscopy [26]. Nevertheless, fluorescence microscopy combined with fluorescence protein fusion [8] has yet to be exploited for investigating the spatiotemporal distribution and secretion of cellulase, a powerful strategy capable of providing direct and clear visualization.

In this study, three major cellulase components BGL, CBH, and CMC fused to red fluorescence protein DsRed, respectively, were successfully expressed in *T. reesei* RUT-C30, resulting in recombinant strains RBGL, RCBH, and RCMC. This allowed the visualization of spatiotemporal distribution and secretion of cellulase in living *T. reesei* by confocal laser scanning microscopy (CLSM) and fluorescence microplate reader. Moreover, the kinetics of cellulase production and the effect of BFA on cellulase production were investigated in these *T. reesei* mutants.

## Materials and methods

### Strains, plasmids, and culture conditions

*Escherichia coli* DH5α was used for plasmid construction and propagation. *T. reesei* RUT-C30 (CICC 13052) was utilized for RNA and DNA extraction, and plasmid transformation. *E. coli* DH5α and *Agrobacterium tumefaciens* AGL-1 were grown in LB medium with 220 rpm at 37 °C and 28 °C, respectively. *T. reesei* was grown at 28 °C with 200 rpm on potato dextrose agar (PDA) plate for conidial culture. Plasmid pDht/sk was given as a gift from Professor Zhihua Zhou from Key Laboratory of Synthetic Biology, Shanghai [27]. The primers used in this study are listed in Additional file 1: Table S1. All chemicals used in this study were purchased from Sigma-Aldrich (Sigma-Aldrich, USA).

### Construction of recombinant *T. reesei* strains

The total RNA of *T. reesei* RUT-C30 was extracted with the RNA extraction Kit from Omega Bio-Tek (GA, USA) following the manufacturer’s instruction. The first-strand cDNA was synthesized from RNA of RUT-C30 using HiScript 1st Strand cDNA Synthesis Kit (Vazyme, China). Then, genes *cel3a* (BGL, M419DRAFT_136547/TRIREDRAFT_76672), *cel7a* (CBH, M419DRAFT_125125/TRIREDRAFT_123989), and *cel7b* (CMC, M419DRAFT_5304/TRIREDRAFT_122081) were amplified using cDNA of *T. reesei* RUT-C30 as template with primers listed in Additional file 1: Table S1, and cloned into the backbone of plasmid p-DsRed at *Xba*I loci using the ClonExpress II One-Step Cloning Kit (Vazyme, China) leading to the plasmid pBGL-DsRed, pCBH-DsRed, and pCMC-DsRed (Additional file 1: Fig. S1). Plasmid p-DsRed was constructed by inserting gene DsRed into the plasmid pDht/sk at XbaI loci. The XbaI loci at the end of gene DsRed on plasmid p-DsRed was mutated, leaving single XbaI for further cloning. These four plasmids were transformed into *T. reesei* RUT-C30 by *A. tumefaciens*-mediated transformation (AMT) method [28] using hygromycin B as a marker, resulting in recombinant strains RBGL, RCBH, RCMC, and RD, respectively. After transferring and selection, single spore colonies of these mutants were isolated for further study. The insertion of targeted genes into *T. reesei* genome was confirmed by PCR amplification using primers shown in Additional file 1: Table S2 and the results are shown in Additional file 1: Fig. S2.

### Shake flask cultivation

For cellulase production, the conidial suspension (0.5 mL, 10^7^/mL) was inoculated into a 50 mL Erlenmeyer flask-containing 10 mL sabouraud dextrose broth (SDB) and incubated for 48 h at 28 °C with 200 rpm. The culture was then transferred into a 250 mL flask-containing 50 mL TMM media (pH 6.0) [29] with 2% (w/t) cellulose or other carbon sources as indicated in the context for cellulase production, and cultivated at 28 °C with 200 rpm. 0.5 mL cell culture was sampled at different time points as indicated in the text, and centrifuged at 14,000*g* for 10 min at 4 °C. The supernatants were filtrated with 0.45 µm filter membrane and stored at − 80 °C for cellulase activity assays and fluorescence intensity measurement. The TMM medium was prepared as followed (all concentrations in g/L unless otherwise noted): urea, 1.00; (NH_4_)_2_SO_4_, 4.00; KH_2_PO_4_, 6.59; FeSO_4_ * 7H_2_O, 0.005; MnSO_4_ * H_2_O, 0.0016; ZnSO_4_ * 7H_2_O, 0.0014; CoCl_2_ * 6H_2_O, 0.002; MgSO_4_, 0.60; CaCl_2_, 0.60; Tween-80, 0.0186% (v/v); tryptone, 0.75; yeast extract, 0.25; maleic acid, 11.6; cellulose 2% (w/v), or other carbon sources as indicated in the context. The pH of TMM was adjusted to 6.0 by NaOH.

For BFA treatment, the cultures grown in TMM + 2% cellulose for 4 days were further diluted 1/10 into fresh TMM + 2% cellulose + 50 μg/mL BFA. The diluted cell cultures were cultivated at 28 °C with 200 rpm. Samples were taken at different time points as indicated in the text, and centrifuged at 8000 rpm for 30 min for the separation of the mycelia and the supernatant. The obtained mycelia were used for confocal observation, while the supernatants were utilized for fluorescence intensity detection and cellulase activity assays.

### Analysis methods

Cellulase activity assays were carried out by following our previous studies [30–32]. Fluorescence intensity measurement of the filtered cell culture supernatant was performed using a Hitachi 2000 fluorescence spectrophotometer (Hitachi Ltd., Japan) with a 650 nm emission filter. The excitation wavelength was 540 nm.

### Confocal imaging of fungal mycelia

Mycelia of *T. reesei* RUT-C30 and its derivatives were loaded onto a glass slide, covered with a cover glass, and observed on an inverted confocal laser scanning microscope SP8 (Leica, Germany) with a 100 × 1.4 NA oil-immersion objective. The red fluorescence protein DsRed was imaged with the excitation wavelength of 552 nm and the emission wavelength of 570–700 nm. The green fluorescence of the cell membrane/wall dye GC-Chol-PEG-FITC developed in our lab [33] was observed at 500–550 nm with the excitation length of 488 nm.

### Preparing protoplasts of *T. reesei* RBGL for confocal imaging

The preparation of *T. reesei* protoplast performed according to the published method [34]. Briefly, 5 mL cell cultures of *T. reesei* RBGL grown in TMM + 2% cellulose for 72 h were centrifuged for 20 min at 8000 rpm to obtain the fungal mycelium, which was resuspended with 6 mL of lysing enzymes from *Trichoderma harzianum* (L1412, Sigma-Aldrich, USA). 675 mg lysing enzyme was dissolved in 15 mL 1.2 M MgSO_4_/10 mM sodium phosphate, pH5.8. The mixture was incubated at 25 °C with 90 rpm for 3–4 h. The release of protoplasts during the incubation was monitored on a microscope by counting the protoplasts using a counting chamber, while the fluorescence of the protoplast sample was detected on an inverted confocal laser scanning microscope SP8 with a 100× oil-immersion objective.

### Confocal imaging of fungal ER and Golgi

ER-Green (DIOC6(3)) (KeyGEN BioTECH Co. Ltd., China) was prepared as a stock solution of 1 mM in DMSO and stored at − 20 °C in dark. Fungal mycelia were washed with Hank’s balanced salt solution (HBSS) buffer for three times and resuspended in 1 mL HBSS buffer. The pre-warmed ER-Green stock solution at 37 °C was added into the suspension at the ratio of 1:1000 and incubated at 37 °C for 20 min under dark condition. Then, the culture was washed with HBSS for two times and resuspended in HBSS buffer before being detected on an inverted confocal laser scanning microscope SP8 with a 100× oil-immersion objective. The excitation wavelength was 488 nm, and the emission was checked in the range of 498–600 nm.

Golgi-Green (C6-NBD-CERAMIDE) (KeyGEN BioTECH Co. Ltd., China) was used to stain the Golgi. Cell culture was washed with HBSS buffer for three times before being resuspended in HBSS buffer. Golgi-Green (5 µM) and BSA (5 µM) were added into the cell suspension followed by incubation at 4 °C for 30 min. The cell suspension was washed with pre-cooled HBSS and incubated at 37 °C for 30 min. Then, the samples were washed with HBSS for two times and observed by fluorescence microscopy as performed for ER staining.

## Results

### Cellulase is located throughout the whole hyphal cell with tip-high gradient, but not septum

**Fig. 1:**
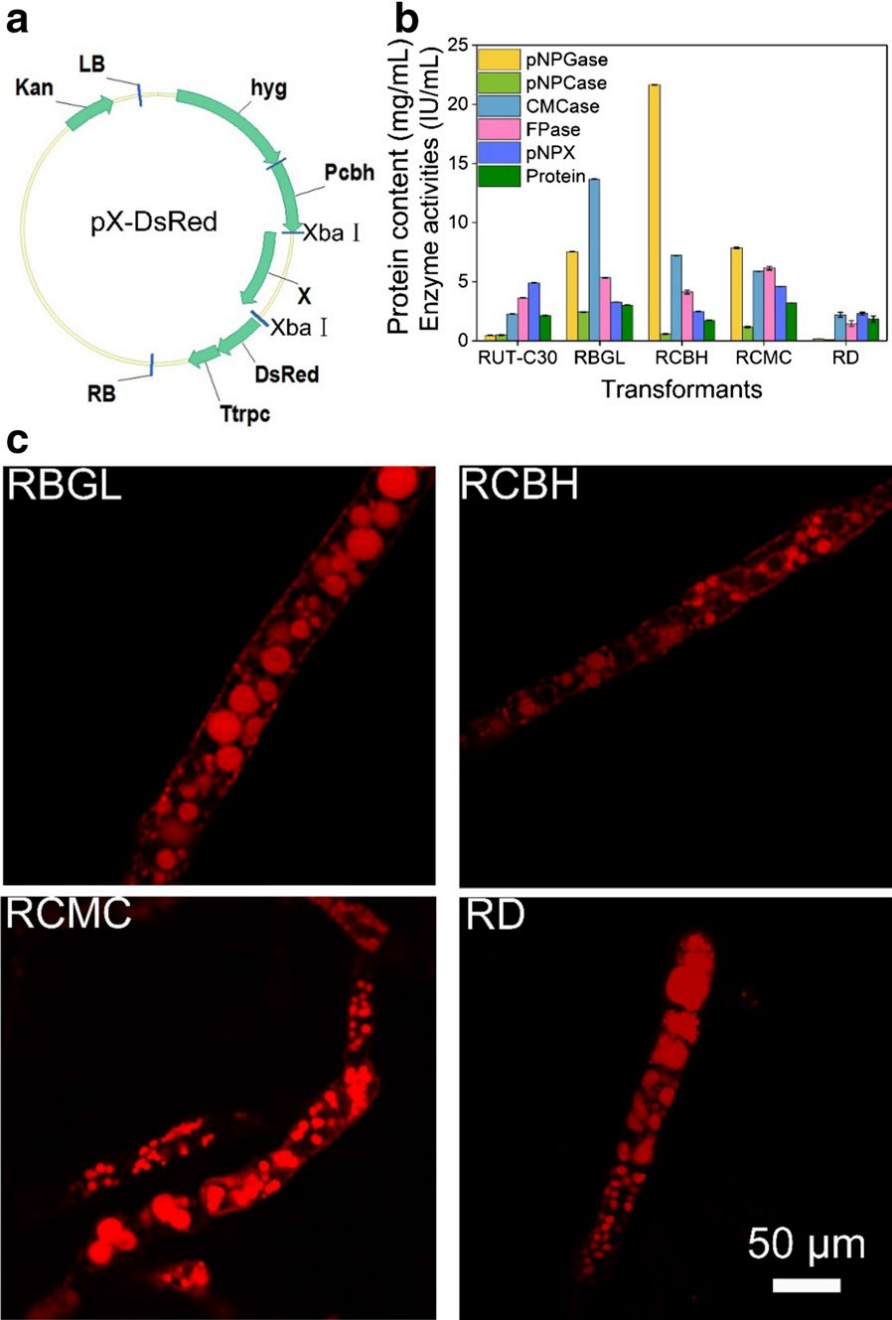
**a** Schematic illustration of the plasmid pX-DsRed (X = CEL3A, CEL7A, or CEL7B). Kan: kanamycin resistance; LB, left border of the binary vector; RB, right border of the binary vector; Pcbh, a modified CBH1 promoter; Ttrpc, *Aspergillus nidulans* trpC terminator; hyg, hygromycin B phosphotransferase gene. **b** Cellulase activities of *T. reesei* RUT-C30, RBGL, RCBH, RCMC, and RD grown on TMM + 2% cellulose for 5 days. pNPGase: the β-glucosidase activity; pNPCase: the CBH activity; CMCase: the CMC activity; FPase: the filter paper activity. Data are represented as the mean of three independent experiments and error bars express the standard deviations. **c** Observation of the localization of BGL-DsRed, CBH-DsRed, CMC-DsRed, and DsRed in *T. reesei* strains RBGL, RCBH, RCMC, and RD, respectively, grown on cellulose for 5 days by STED

Expression of cellulases BGL, CBH, and CMC as a fusion protein with red fluorescence protein DsRed under a modified CBH1 promoter (Fig. 1a) in *T. reesei* RUT-C30 was carried out, leading to recombinant strains RBGL, RCBH, and RCMC, respectively. Expression of DsRed alone in strain RUT-C30 was also performed as a control, which was designated as strain RD. We obtained 5, 2, 10, and 2 transformants for RBGL, RCBH, RCMC, and RD, respectively. The cellulase activity of these mutant strains was measured on day 5 using cellulose as the only carbon source (Additional file 1: Fig. S3), while confocal laser scanning microscopy (CLSM) was applied to check whether there was red fluorescence inside the cells. RBGL-1, RCBH-6, RCMC-8, and RD-1 were chosen for further study according to two rules: their cellulase activities were not affected notably in comparison with the parent strain RUT-C30 (Fig. 1b and Additional file 1: Fig. S3) and red fluorescence was observed in the fungal cells under CLSM (Fig. 1c and Additional file 1: Fig. S4). From here, strains RBGL-1, RCBH-6, RCMC-8, and RD-1 were named as RBGL, RCBH, RCMC, and RD, respectively.

The cellular distribution of the three major cellulases was investigated by stimulated emission depletion microscopy (STED) using recombinant strains cultured in TMM containing 2% cellulose for 5 days (Fig. 1c). Significant red fluorescent punctuates were observed throughout the cytoplasm of strains RBGL, RCBH, and RCMC, showing that all three cellulases BGL CBH, and CMC were present abundantly in secretory vesicles/vacuoles of the cytoplasm (Fig. 1c). Interestingly, we also observed red fluorescence on the cell membrane/wall of the three recombinant strains RBGL, RCBH, and RCMC, while no fluorescence was found on the cell membrane of the control strain RD. In line with this, all the hyphae of stains RBGL, RCBH, and RCMC displayed red fluorescence under low magnification due to the distribution of the fusion proteins BGL-DsRed, CBH-DsRed, and CMC-DsRed on cell membrane/wall (Additional file 1: Fig. S4), while RD did not as the fluorescence protein DsRed alone cannot located on the cell membrane/wall. It seems that the three major cellulases BGL, CBH, and CMC were all located on the cell membrane/wall, which is consistent with previous findings [14, 35–37].

**Fig. 2:**
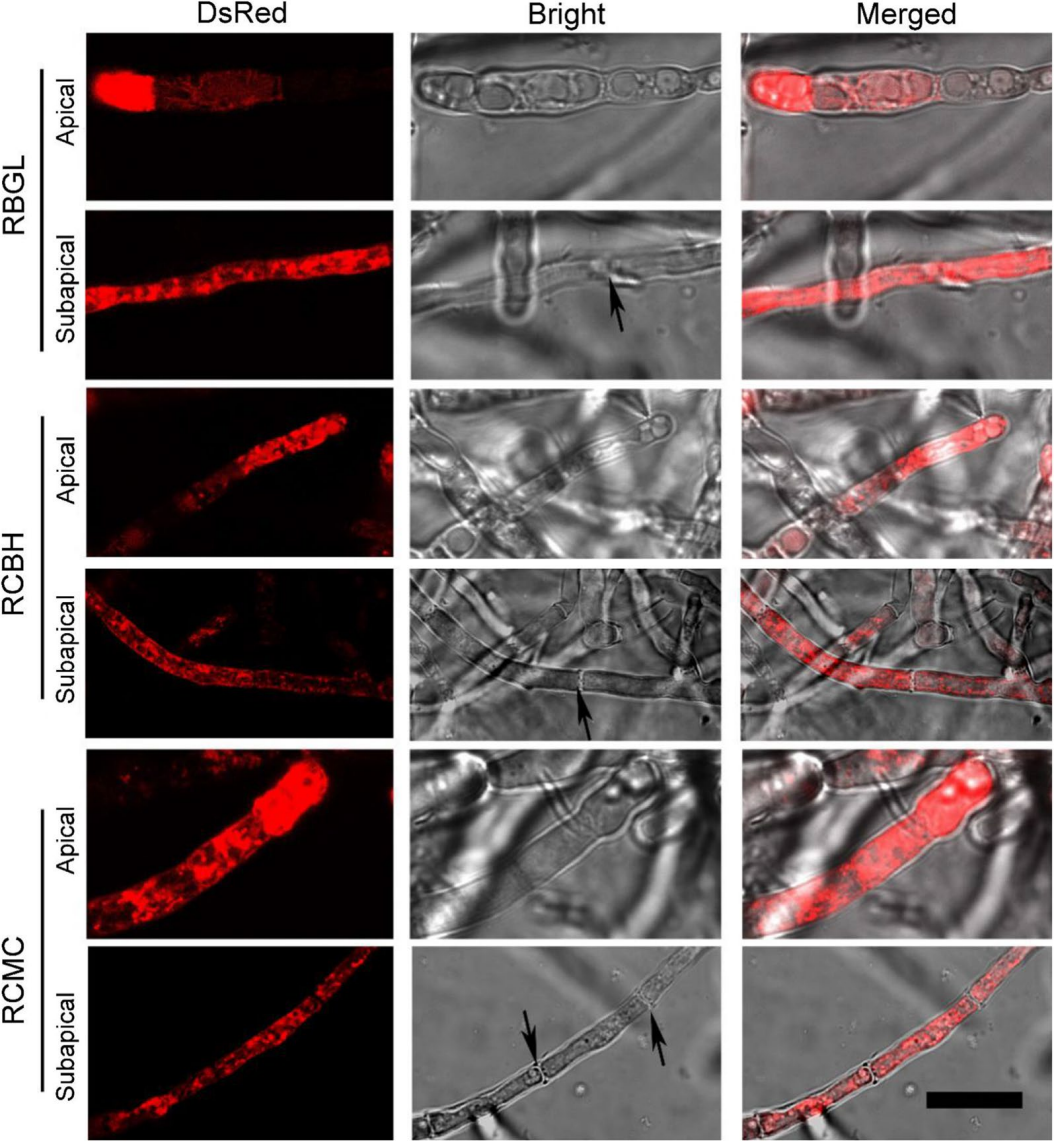
Cellular localization of BGL-DsRed, CBH-DsRed, and CMC-DsRed at the apical and subapical regions of the recombinant strains RBGL, RCBH, and RCMC, respectively, grown in TMM + 2% cellulose for 4 days. The black arrowheads indicate the septum (Scale bar = 15 μm)

In all three recombinant strains, strong fluorescence was found both at the hyphal tip and distal hyphae regions with brighter fluorescence at the hyphal apexes (Fig. 2). It is probably that all three cellulases accumulated abundantly at the Spitzenkorper of the hyphal tips, indicating that the bulk flow accompanied with polarized apical growth of hyphae is main secretory pathway for cellulase. However, no red fluorescence was obtained at the septa of the three recombinant strains, suggesting that cellulase was not located on the hyphae septa. That all three tested cellulases were distributed throughout the whole hyphae except septum would enable their effective excretion from the fungal cells. This may partially explain the high extracellular cellulase production of RUT-C30. It seems that a complementary mechanism for protein secretion to the bulk flow accompanied with polarized apical growth of hyphae is adapted by *T. reesei* to release cellulase into the culture medium.

### BGL is located on cell membrane but not cell wall

**Fig. 3:**
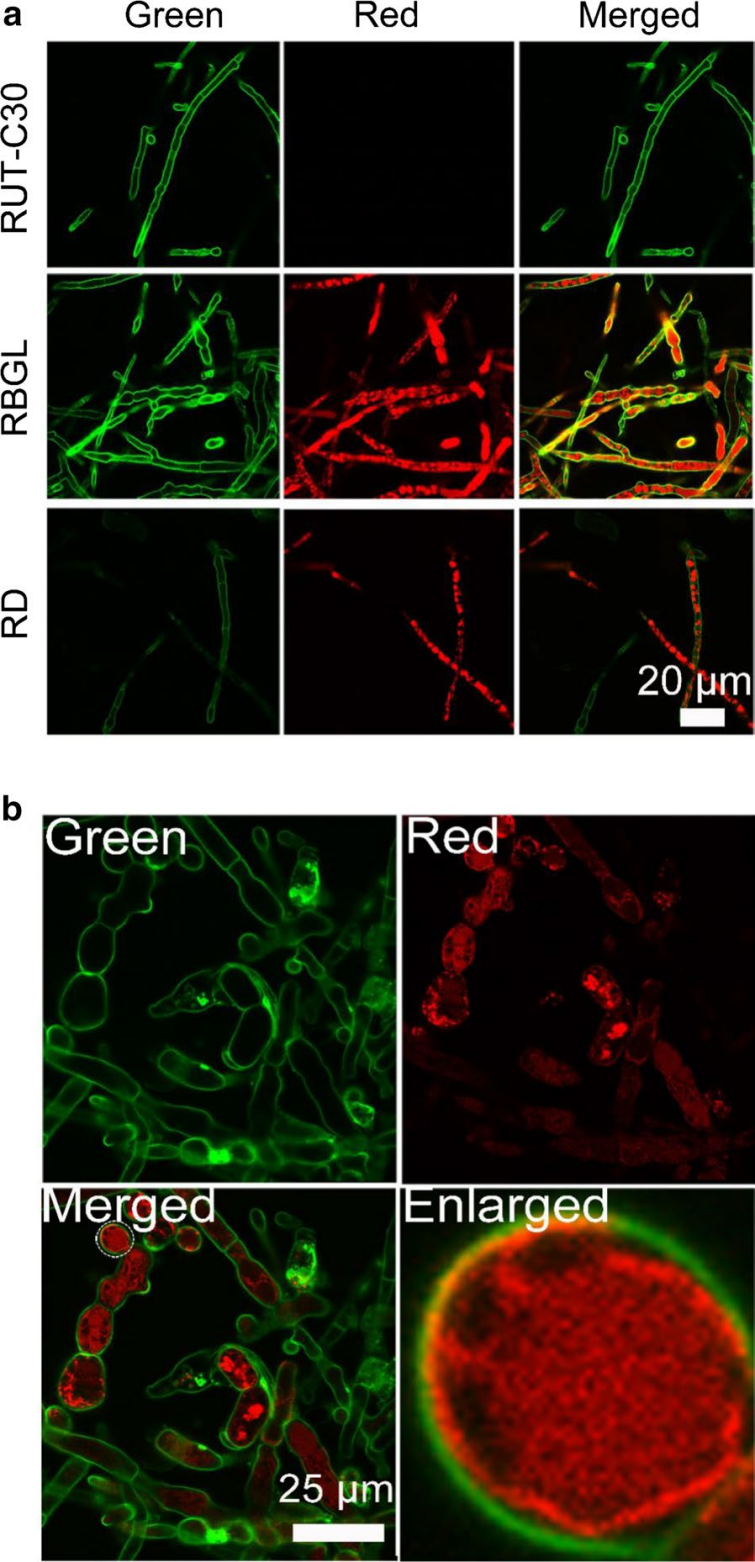
**a** Confocal images of strains RUT-C30, RBGL, and RD (Red) cultured for 5 days and stained with cholesterol-PEG-FITC (Green). **b** Confocal images of the protoplasts of strain RBGL (Red) stained with cholesterol-PEG-FITC (Green)

To further confirm that BGL is distributed on cell membrane/wall, cholesterol-PEG-FITC, a cell membrane/wall dye synthesized in our lab [33], was employed to stain the cell membrane of *T. reesei* RUT-C30, RBGL and RD (Fig. 3a). The red fluorescent signal on cell membrane/wall of strain RBGL colocalized well with green fluorescence of cholesterol-PEG-FITC, resulting in yellow fluorescence on the cell membrane/wall of strain RBGL as observed in the merged figure of strain RBGL. In contrast, no yellow fluorescence was observed for strain RD, although green fluorescence was found on cell membrane/wall and red fluorescence was obtained in the cytoplasm. This further evidenced that protein BGL was localized on the cell membrane/wall. Nevertheless, whether BGL is on cell membrane or on cell wall is still unknown, as it is so difficult to differentiate cell membrane from cell wall. Therefore, protoplast of strain RBGL cultivated in TMM + 2% cellulose for 3 days was obtained and stained with cholesterol-PEG-FITC (Fig. 3b). In the plasmolysis cells of strain RBGL (Fig. 3b), the overlap of cholesterol-PEG-FITC with BGL-DsRed was reduced notably with only a few regions displaying yellow fluorescence as compared to the untreated mycelium cells of strain RBGL (Fig. 3a). In the enlarged image of one plasmolysis cell which was undergoing plasmolysis, the red fluorescence was being separated from green fluorescence along with the dividing of the cell membrane from the cell wall, while yellow fluorescence was still observed on the regions where plasmolysis did not occur. This result strongly supports that protein BGL is distributed on cell membrane rather than cell wall.

### The cellular distribution of cellulase

**Fig. 4:**
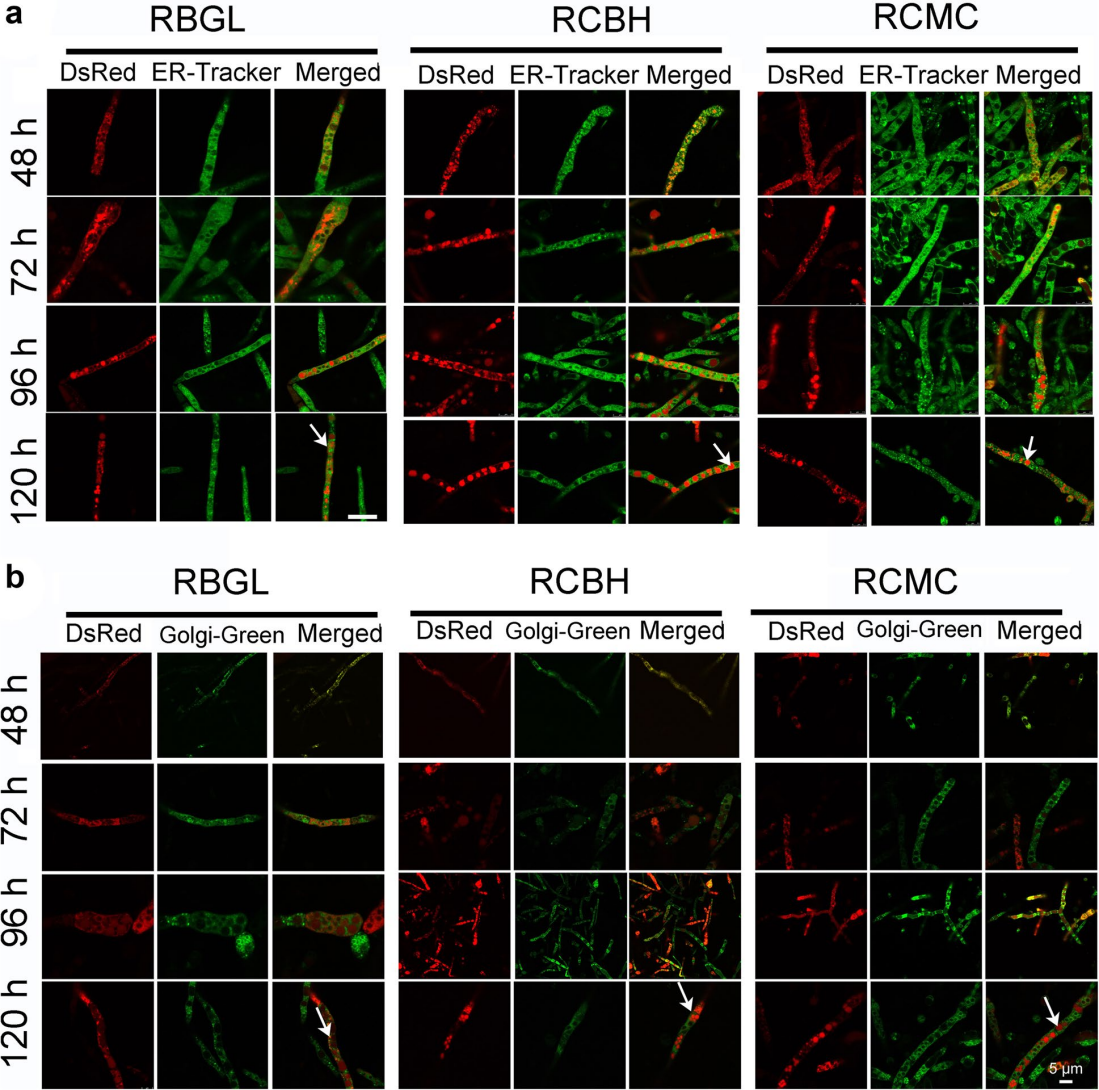
Confcal images of strains RBGL, RCBH, and RCMC stained with ER-Tracker (**a**) and Golgi-Green (**b**), respectively. The white arrowheads point out the vacuoles

To know exactly the subcellular distribution of cellulases BGL, CMC, and CBH inside the hyphae cells, recombinant strains RBGL, RCBH, and RCMC grown at different time points during the cellulase production was treated with ER-tracker and Golgi-Green to stain ER (Fig. 4a) and Golgi (Fig. 4b), respectively. Green fluorescence from ER-tracker was observed throughout the whole hyphae, suggesting that *T. reesei* has well-developed ER network, which is consistent with the previous finding that the growing mycelia of *T. reesei* RUT-C30 were richly endowed with ER [38]. On the other hand, green fluorescence was observed when the three recombinant strains were exposed to Golgi-Green for Golgi-staining, demonstrating that *T. reesei* has Golgi structure as found by the previous study [39]. Nevertheless, this result contradicts with previous reports that no Golgi-like bodies were found in *T. reesei* RUT-C30 [26, 38]. Partial overlap of all three tested cellulase components BGL-DsRed, CBH-DsRed, and CMC-DsRed with ER or Golgi was found, as indicated by the observation of yellow fluorescence (Fig. 4a, b). However, the co-localization of BGL-DsRed, CBH-DsRed, and CMC-DsRed with the Golgi structure was poor (Fig. 4b), which coincided with the early finding that the labeling of endoglucanase I in Golgi-like structure was bad [39]. Interestingly, BGL-DsRed, CBH-DsRed, and CMC-DsRed accumulated largely in the lumen of globular vacuoles in addition to ER and Golgi (Fig. 4), which was in line with the result obtained by STED (Fig. 1c). Both xylanase II (XYN II) [26] and endoglucanase I [39] have been found in vacuoles. It is supposed that the cellulase secretion by *T. reesei* might involve vacuoles [26, 38]. Another hypothesis is that these hydrolytic enzymes are stored in the vacuoles in a poorly active form to avoid the deleterious effect of the hyperproduction of cellulase in *T. reesei* cells [38]. However, more evidences from experiments are required to support these views.

### Monitoring kinetic of cellulase production

**Fig. 5:**
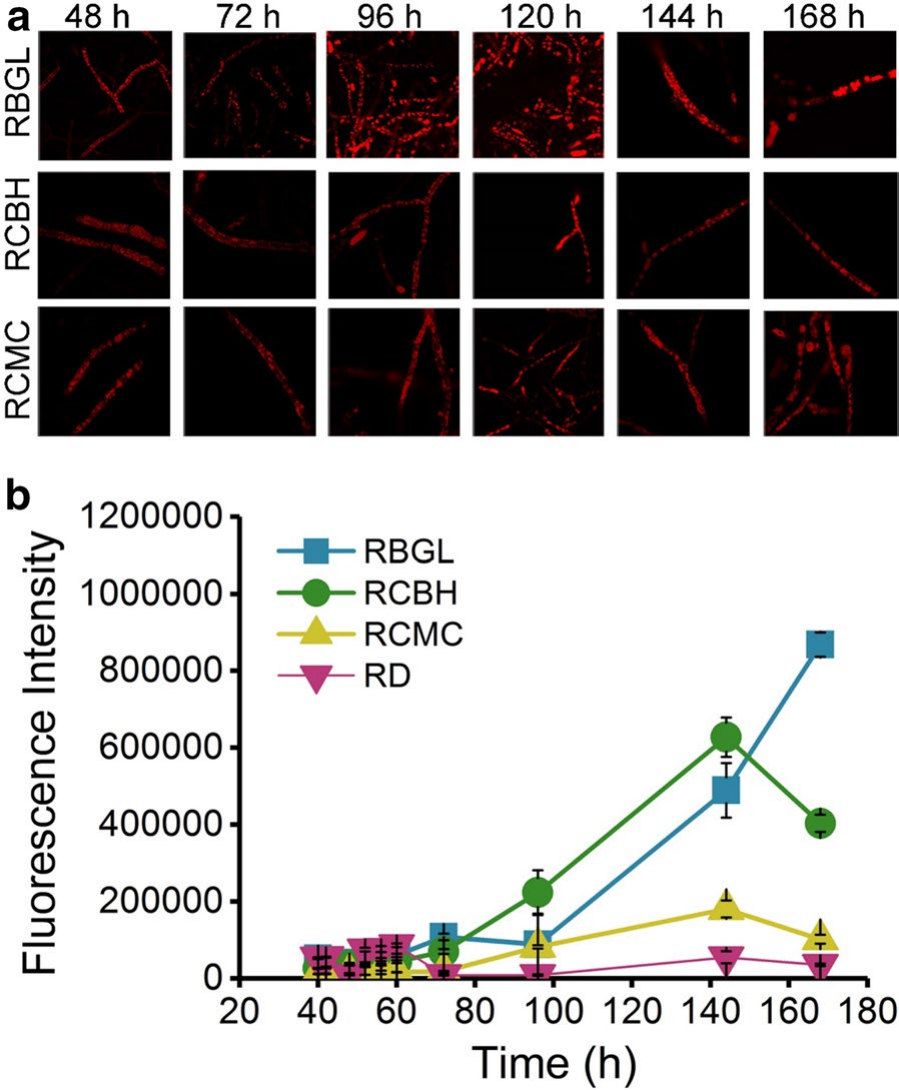
Confocal images of mycelia (**a**) and fluorescence intensity of the supernatants (**b**) from the recombinant strains RBGL, RCBH, and RCMC during the cellulase fermentation. The excitation wavelength was 552 nm

Using the recombinant strains RBGL, RCMC, and RCBH, real-time monitor of cellulase production in live *T. reesei* was performed inside the cells by CLSM (Fig. 5a) and in the supernatant via spectroscopic analysis (Fig. 5b). At 48 h, red fluorescence was clearly observed in the cytoplasm of the hyphae cells of the three mutants, suggesting that BGL-DsRed, CBH-DsRed, and CMC-DsRed were expressed (Fig. 5a). The red fluorescence was strong in the cytoplasm of the three mutant hyphae cells at different time points during the whole fermentation process (from 48 to 168 h) with an increased fluorescence intensity observed from 96 to 144 h (Fig. 5a). In agreement with this finding, the fluorescence intensity in the supernatant of strain RCMC and RCBH remained almost unchanged from 48 to 72 h, followed by a notable increase from 72 to 144 h, while the fluorescence intensity in the supernatant of strain RBGL was not remarkably increased until 96 h. No fluorescence was detected in the supernatant of strain RD during the whole process. Obviously, there is good correlation between the cellulase expression in the hyphae and the cellulase secretion into the culture medium. The effective secretion of CMC and CBH began in the midterm of the fermentation, while the efficient release of BGL started in the late stage.

### BFA inhibits secretion of total cellulase in *T. reesei*

**Fig. 6:**
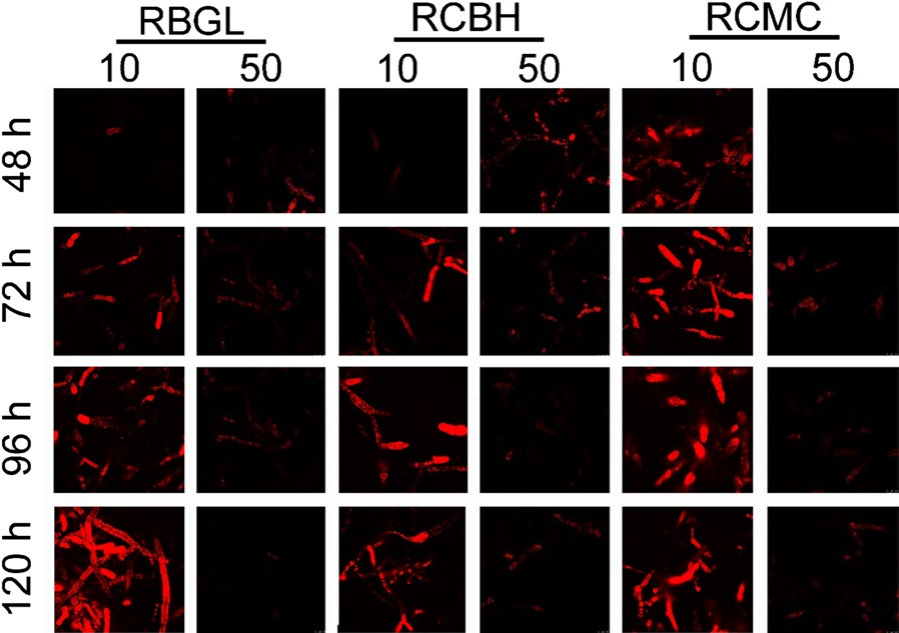
Confocal images of recombinant strains RBGL, RCBH and RCMC cultured in TMM + 2% cellulose + 10 or 50 μg/mL BFA for 48, 72, 96 and 120 h

**Fig. 7:**
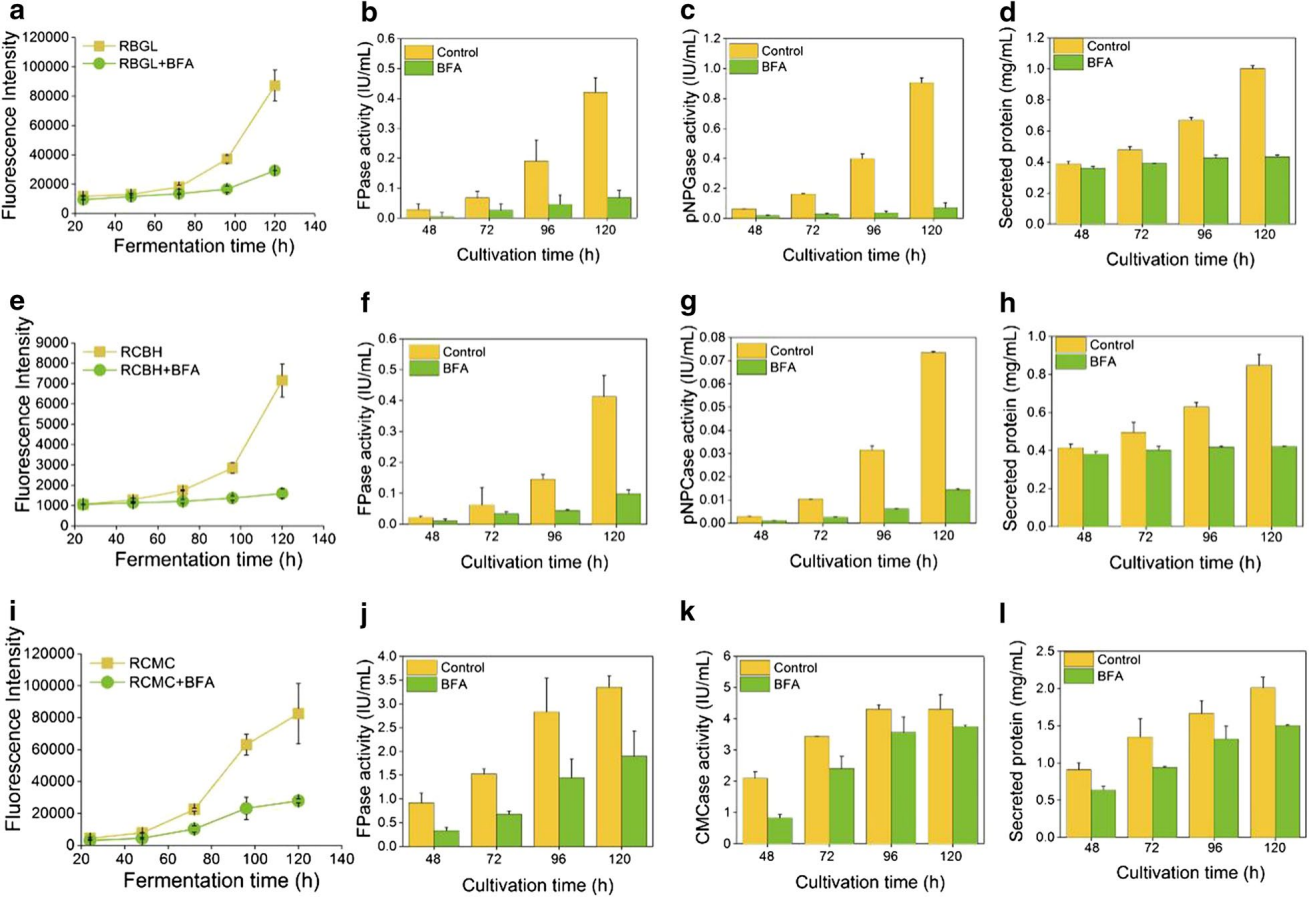
Fluorescence intensity (**a**, **e**, **i**), FPase (**b**, **f**, **j**), pNPGase activity (**c**), pNPCase activity (**g**), CMCase activity (**k**), and secreted protein concentration (**d**, **h**, **l**) in the culture supernatants of recombinant strains RBGL, RCBH, and RCMC cultured in TMM + 2% cellulose + 10 μg/mL BFA for 48, 72, 96, and 120 h

Recombinant strains RBGL, RCMC, and RCBH were exposed to 10 and 50 μg/mL Brefeldin A (BFA), a compound primarily inhibiting vesicle trafficking between ER and Golgi apparatus in fungi [40]. In the presence of 10 μg/mL BFA, red fluorescence in the mycelia of all three recombinant strains was almost unchanged as compared to the untreated samples (Fig. 6 and Additional file 1: Fig. S5), indicating that the cellulase expression was not affected by 10 μg/mL BFA. Nevertheless, the red fluorescence in the corresponding supernatant samples was neglected and remained almost unchanged throughout the whole cellulase production process (Fig. 7a, e, i), suggesting that cellulase secretion was hampered. Accordingly, the cellulase activities and protein concentration in the supernatant of the three recombinant strains were reduced sharply with the treatment of 10 μg/mL BFA (Fig. 7). When the concentration of BFA was increased to 50 μg/mL, red fluorescence in all three mutants was almost abolished, implying that the cellulase expression was inhibited significantly (Fig. 6). Meanwhile, the red fluorescence in the supernatant of the 50 μg/mL BFA-treated strains RBGL, RCMC, and RCBH stayed unchanged during the cellulase production process (Additional file 1: Fig. S6), suggesting that the cellulase secretion was also retarded by BFA. Taken together, BFA inhibited notably cellulase secretion in *T. reesei,* and even the cellulase expression at high concentration, both leading to notably reduced cellulase production.

## Discussion

Using the industrial strain *T. reesei* RUT-C30 as the parent strain, we have successfully constructed recombinant strains RBGL, RCBH, and RCMC in which cellulase BGL, CBH, and CMC were fused to red fluorescence protein DsRed. In this way, the localization and secretion of cellulase in living *T. reesei* were first investigated directly in a dynamic manner by fluorescence imaging method using CLSM and fluorescence microplate reader. Although it presents a well-established, potent way to analyze the spatiotemporal distribution of protein in microorganism study, fluorescence microscopy combined with fluorescence protein fusion is not fully implicated in the study of *T. reesei* cellulase. Traditionally, the localization and secretion of cellulase has been studied by cellulase activity measurement in subcellular fractionation [23], electron microscopy [13, 38], and immunoelectron microscopy [14, 26, 37]. This is probably due to that the genetic engineering platform of *T. reesei* is not as well developed as the model microorganisms like *Escherichia coli* and *Saccharomyces cerevisiae*. Therefore, it is complicated and cumbersome to perform the strain construction required for fluorescence imaging. It is worth noting that several newly minted of genetic tools for *T. reesei* have been developed in recent years, including CRISPR/Cas9 [41] and I-SceI-mediated genetic box [42, 43]. With these advances of genetic tools in *T. reesei*, the fluorescence imaging can definitely become a powerful and common way to investigate spatiotemporal localization and secretion of *T. reesei*, as demonstrated in this study.

Although the mechanism of cellulase production in fungi has been extensively studied [44], few research on spatiotemporal distribution of cellulase in fungi has been reported, retarding the deeper understanding of the molecular mechanism behind the fungal cellulase production. Here, we found that three major cellulase components BGL, CMC, and CBH were distributed throughout the whole fungal hypha except septa, which is consistent with previous findings [14, 35–38, 45, 46]. By indirect immunofluorescence microscopy and labeled cryosections, protein CBHI was found throughout the whole mycelium including vesicles and hyphal walls, but not on septa [14]. Endoglucanases were found in both hyphal tips and hyphal walls as found by immunoelectron microscopy [37]. Accumulation of CMC in cytosol, vacuoles, and ER of *T. reesei* was observed in another study using electron microscopy [38]. BGLs have been reported to be extracellular [45], plasma-membrane-bound [36], and intracellular [46]. However, these early studies are based on processed fungal cell samples without studying the localization of cellulase in living *T. reesei* in real time and in a dynamic way, which instead was achieved in this study. Moreover, for the first time, we demonstrated that BGL1 was located on cell membrane but not cell wall. Owing to the inefficient separation of cell wall and cell membrane by the used techniques, the three major cellulases CMC, CBHI, and BGLI were generally reported to be cell-wall-bounded [14, 35–37].

BFA is an inhibitor of the traditional ER–Golgi secretory pathway, generally utilized to prevent vesicle budding from the Golgi. In many types of cells, the structure of the Golgi compartment was damaged in the presence of BFA [47]. The reported targets of BFA include the guanine nucleotide exchange factors of ARF proteins required for transport vesicle coat formation [48–50], and the BARS proteins that are linked to the integrity of Golgi structure [51]. Our finding that BFA totally blocked the expression and secretion of cellulase in *T. reesei* is in accordance with the early results. The short-term effect of BFA on the cellulase CBHI synthesis and transport in *T. reesei* was investigated at both protein and mRNA level [52]. The half-life of the *cbh1* mRNA, the rate of CBHI synthesis, and CBHI transport were reduced significantly in the BFA-treated cultures, leading to that only a minute amount of the labeled CBHI with [^35^S] methionine was detectable in the culture medium at the late stages of the labeling experiment.

The tip-high gradient of intracellular cellulase (Fig. 2), the co-localization of intracellular cellulase with ER and Golgi (Fig. 4), and the complete inhibition of cellulase production by BFA (Fig. 7) all support the proposition that the cellulase secretion is majorly via the classical ER–Golgi secretory pathway. Meanwhile, the localization of cellulase throughout the whole fungal mycelia (Fig. 5) except septa, and the abundant accumulation of cellulase in vacuoles (Figs. 1c, 4) demonstrate that there are probably other unconventional protein secretion pathways for cellulase secretion. Recent studies unraveled several alternative protein secretory pathways to the conventional secretory pathway in fungi, such as Golgi-independent secretion which was BFA-insensitive [9], secretion independent of both ER and Golgi apparatus [10], and extracellular vesicle secretion [11]. Nevertheless, whether and how these unconventional pathways are involved in cellulase requires further extensive study.

## Conclusion

For the first time, the spatiotemporally localization and secretion of cellulase was monitored directly in live *T. reesei*. From *T. reesei* RUT-C30, we have successfully constructed recombinant *T. reesei* RBGL, RCBH, and RCMC, which expressed DsRED-fused versions of BGL, CBH, and CMC, namely BGL-DsRed, CBH-DsRed, and CMC-DsRed. In these recombinant strains, BGL, CBH, and CMC was localized throughout the whole fungal mycelia with enormous accumulation at the growing apices, including ER, Golgi, vacuoles, and cell membrane/wall but not septa. In addition, the major secretion of CBH and CMC begun much earlier than that of BGL. In the presence of BFA, the cellulase expression and secretion was severely inhibited in *T. reesei*, resulting in neglectable fluorescence intensity and cellulase activities. All these results further support the view that cellulase excretion mainly occurs via the conventional ER–Golgi secretory pathway, as evidenced by the abundant accumulation of intracellular cellulase at the advancing tips (Fig. 2), the localization of intracellular cellulase in ER and Golgi (Fig. 4), and the inhibition of cellulase secretion by BFA (Figs. 6, 7), but might be assisted through unconventional protein secretion pathways as it was found that cellulase distributed throughout the whole fungal mycelia (Fig. 5a) except septa with the notable accumulation in vacuoles (Figs. 2, 4).

## Additional file

**Additional file 1: Fig. S1.** Sequences of plasmid pX-DsRed. Pcbh, Ttrpc, hyg, linker, and XbaI were represented in light green, pink, dark green, blue and light red respectively; CEL3A, CEL7A, CEL7B and DsRed were marked in purple, yellow, dark red and grey, respectively; The LB and RB of the binary vector were underlined and in bold. **Fig. S2.** PCR conformation results for RUT-C30 and the transformants RCMC, RCBH and RBGL. **Fig. S3.** Cellulase activities of recombinant *T. reesei* strains RBGL (A), RCBH (B), RCMC (C), and RD (D). The mutants were grown in TMM + 2% cellulose for 5 days. pNPGase: the β-glucosidase activity; pNPCase: the CBH activity; CMCase: the CMC activity; FPase: the filter paper activity. Data are represented as the mean of three independent experiments and error bars expressed the standard deviations. **Fig. S4.** CLSM imaging of RBGL, RCBH, RCMC, and RD cultivated in TMM + 2% cellulose for 5 days using 10 × objective. **Fig. S5.** Confocal images of recombinant strains RBGL, RCBH and RCMC cultured in TMM + 2% cellulose + 100 μL or 500 μL DMSO for 48, 72, 96 and 120 h. **Fig. S6.** Fluorescence intensity (A, C, and E), and secreted protein concentration (B, D, and F) in the culture supernatants of recombinant strains RBGL, RCBH and RCMC cultured in TMM + 2% cellulose + 50 μg/mL BFA for 48, 72, 96 and 120 h. **Table S1.** PCR primers for plasmid construction. **Table S2.** PCR primers for confirmation of transformants.

## Abbreviations

pNPGase: the β-glucosidase activity
pNPCase: the CBH activity
CMCase: the CMC activity
FPase: the filter paper activity
pNPXase: the β-xylosidase activity
AMT: *Agrobacterium tumefaciens*-mediated transformation
PDA: potato dextrose agar
SDB: sabouraud dextrose broth
TMM: *Trichoderma* minimal medium
