# Supplementary material for: Tracking localization and secretion of cellulase spatiotemporally and directly in living Trichoderma reesei
Source: Biotechnol Biofuels. 2019 Aug 20;12:200. doi: 10.1186/s13068-019-1538-0 (PMC6700804; doi:10.1186/s13068-019-1538-0)
Supplement: Supplementary file 1 — Additional file 1: Fig. S1. Sequences of plasmid pX-DsRed. Pcbh, Ttrpc, hyg, linker, and XbaI were represented in light green, pink, dark green, blue and light red respectively; CEL3A, CEL7A, CEL7B and DsRed were marked in purple, yellow, dark red and grey, respectively; The LB and RB of the binary vector were underlined and in bold. Fig. S2. PCR conformation results for RUT-C30 and the transformants RCMC, RCBH and RBGL. Fig. S3. Cellulase activities of recombinant T. reesei strains RBGL (A), RCBH (B), RCMC (C), and RD (D). The mutants were grown in TMM + 2% cellulose for 5 days. pNPGase: the β-glucosidase activity; pNPCase: the CBH activity; CMCase: the CMC activity; FPase: the filter paper activity. Data are represented as the mean of three independent experiments and error bars expressed the standard deviations. Fig. S4. CLSM imaging of RBGL, RCBH, RCMC, and RD cultivated in TMM + 2% cellulose for 5 days using 10 × objective. Fig. S5. Confocal images of recombinant strains RBGL, RCBH and RCMC cultured in TMM + 2% cellulose + 100 μL or 500 μL DMSO for 48, 72, 96 and 120 h. Fig. S6. Fluorescence intensity (A, C, and E), and secreted protein concentration (B, D, and F) in the culture supernatants of recombinant strains RBGL, RCBH and RCMC cultured in TMM + 2% cellulose + 50 μg/mL BFA for 48, 72, 96 and 120 h. Table S1. PCR primers for plasmid construction. Table S2. PCR primers for confirmation of transformants. [file 13068_2019_1538_MOESM1_ESM.docx]

**Additional file 1:**

aacattaacgtttacaatttcgcgccattcgccattcaggctgcgcaactgttgggaagggcgatcggtgcgggcctcttcgctattacgccagctggcgaaagggggatgtgctgcaaggcgattaagttgggtaacgccagggttttcccagtcacgacgttgtaaaacgacggccagtgaattgtaatacgactcactatagggcgaattgggtaccgggccccccctcgaggtcgacggtatcgataagcttgatatcgaattcctgcagcccgggggatccgcgcaattaaccctcactaaagggaacaaaagctggagctccaccgcggtggcggccgcgacgttaactgatattgaaggagcatttttgggcttggctggagctagtggaggtcaacacatcaatgctattttggtttagtcgtccaggcggatcacaaaatttgtgtcgtttgacaagatggttcatttaggcaactggtcagatcagcccacttgtaagcagtagcggcggcgctcgaagtgtgactcttattagcagacaggaacgaggacattattatcatctgctgcttggtgcacgataacttgtgcgtttgtcaagcaaggtaagtgaacgacccggtcataccttcttaagttcgcccttcctccctttatttcagattcaatctgacttacctattctacccaagcatcgatatgaaaaagcctgaactcaccgcgacgtctgtcgagaagtttctgatcgaaaagttcgacagcgtctccgacctgatgcagctctcggagggcgaagaatctcgtgctttcagcttcgatgtaggagggcgtggatatgtcctgcgggtaaatagctgcgccgatggtttctacaaagatcgttatgtttatcggcactttgcatcggccgcgctcccgattccggaagtgcttgacattggggaattcagcgagagcctgacctattgcatctcccgccgtgcacagggtgtcacgttgcaagacctgcctgaaaccgaactgcccgctgttctgcagccggtcgcggaggccatggatgcgatcgctgcggccgatcttagccagacgagcgggttcggcccattcggaccgcaaggaatcggtcaatacactacatggcgtgatttcatatgcgcgattgctgatccccatgtgtatcactggcaaactgtgatggacgacaccgtcagtgcgtccgtcgcgcaggctctcgatgagctgatgctttgggccgaggactgccccgaagtccggcacctcgtgcacgcggatttcggctccaacaatgtcctgacggacaatggccgcataacagcggtcattgactggagcgaggcgatgttcggggattcccaatacgaggtcgccaacatcttcttctggaggccgtggttggcttgtatggagcagcagacgcgctacttcgagcggaggcatccggagcttgcaggatcgccgcggctccgggcgtatatgctccgcattggtcttgaccaactctatcagagcttggttgacggcaatttcgatgatgcagcttgggcgcagggtcgatgcgacgcaatcgtccgatccggagccgggactgtcgggcgtacacaaatcgcccgcagaagcgcggccgtctggaccgatggctgtgtagaagtactcgccgatagtggaaaccgacgccccagcactcgtccgagggcaaaggaatagagtagatgccgaccggatcgatccacttaacgttactgaaatcatcaaacagcttgacgaatctggatataagatcgttggtgtcgatgtcagctccggagttgagacaaatggtgttcaggatctcgataagatacgttcatttgtccaagcagcaaagagtgccttctagtgatttaatagctccatgtcaacaagaataaaacgcgttttcgggtttacctcttccagatacagctcatctgcaatgcattaatgcattgactgcaacctagtaacgccttcaggctccggcgaagagaagaatagcttagcagagctattttcattttcgggagacgagatcaagcagatcaacggtcgtcaagagacctacgagactgaggaatccgctcttggctccacgcgactatatatttgtctctaattgtactttgacatgctcctcttctttactctgatagcttgactatgaaaattccgtcaccagccctgactagttttccctgattcagcgtacccgtacaagtcgtaatcactattaacccagactgaccggacgtgttttgcccttcatttggagaaataatgtcattgcgatgtgtaatttgcctgcttgaccgactggggctgttcgaagcccgaatgtaggattgttatccgaactctgctcgtagaggcatgttgtgaatctgtgtcgggcaggacacgcctcgaaggttcacggcaagggaaaccaccgatagcagtgtctagtagcaacctgtaaagccgcaatgcagcatcactggaaaatacaaaccaatggctaaaagtacataagttaatgcctaaaggagtcatataccagcggctaataattgtacaatcaagtggctaaacgtaccgtaatttgccaacggcttgtgggctaacagaagcaacggcaaagccaatcttccaatcgtttgtttcttcactcagtccaatctcagctggtgatcccccaattgggtcgcttgtttgttccggtgaagtgaaagaagacagaggtaagaatgtctgactcggagcgttttgcatacaaccaagggcagtgatggaagacagtgaaatgttgacattcaaggagtatttagccagggatgcttgagtgtatcgtgtaaggaggtttgtctgccgatacgacgaatactgtatagtcacttctgatgaagtggtccatattgaaatgtaagtcggcactgaacaggcaaaagattgagttgaaactgcctaagatctcgggccctcgggccttcggcctttgggtgtacatgtttgtgctccgggcaaatgcaaagtgtggtaggatcgaacacactgctgcctttaccaagcagctgagggtatgtgataggcaaatgttcaggggccactgcatggtttcgaatagaaagagaagcttagccaagaacaatagccgataaagatagcctcattaaacggaatgagctagtaggcaaagtcagcgaatgtgtatatataaaggttcgaggtccgtgcctccctcatgctctccccatctactcatcaactcagatcctccaggagacttgtacaccatcttttgaggcacagaaacccaatagtcaatctagaatgtatcggaagttggccgtcatctcggccttcttggccacagctcgtgctcagtcggcctgcactctccaatcggagactcacccgcctctgacatggcagaaatgctcgtctggtggcacgtgcactcaacagacaggctccgtggtcatcgacgccaactggcgctggactcacgctacgaacagcagcacgaactgctacgatggcaacacttggagctcgaccctatgtcctgacaacgagacctgcgcgaagaactgctgtctggacggtgccgcctacgcgtccacgtacggagttaccacgagcggtaacagcctctccattggctttgtcacccagtctgcgcagaagaacgttggcgctcgcctttaccttatggcgagcgacacgacctaccaggaattcaccctgcttggcaacgagttctctttcgatgttgatgtttcgcagctgccgtgcggcttgaacggagctctctacttcgtgtccatggacgcggatggtggcgtgagcaagtatcccaccaacaccgctggcgccaagtacggcacggggtactgtgacagccagtgtccccgcgatctgaagttcatcaatggccaggccaacgttgagggctgggagccgtcatccaacaacgcgaacacgggcattggaggacacggaagctgctgctctgagatggatatctgggaggccaactccatctccgaggctcttaccccccacccttgcacgactgtcggccaggagatctgcgagggtgatgggtgcggcggaacttactccgataacagatatggcggcacttgcgatcccgatggctgcgactggaacccataccgcctgggcaacaccagcttctacggccctggctcaagctttaccctcgataccaccaagaaattgaccgttgtcacccagttcgagacgtcgggtgccatcaaccgatactatgtccagaatggcgtcactttccagcagcccaacgccgagcttggtagttactctggcaacgagctcaacgatgattactgcacagctgaggaggcagaattcggcggatcctctttctcagacaagggcggcctgactcagttcaagaaggctacctctggcggcatggttctggtcatgagtctgtgggatgattactacgccaacatgctgtggctggactccacctacccgacaaacgagacctcctccacacccggtgccgtgcgcggaagctgctccaccagctccggtgtccctgctcaggtcgaatctcagtctcccaacgccaaggtcaccttctccaacatcaagttcggacccattggcagcaccggcaaccctagcggcggcaaccctcccggcggaaacccgcctggcaccaccaccacccgccgcccagccactaccactggaagctctcccggacctacccagtctcactacggccagtgcggcggtattggctacagcggccccacggtctgcgccagcggcacaacttgccaggtcctgaacccttactactctcagtgcctg(atgcgttaccgaacagcagctgcgctggcacttgccactgggccctttgctagggcagacagtcactcaacatcgggggcctcggctgaggcagttgtacctcctgcagggactccatggggaaccgcgtacgacaaggcgaaggccgcattggcaaagctcaatctccaagataaggtcggcatcgtgagcggtgtcggctggaacggcggtccttgcgttggaaacacatctccggcctccaagatcagctatccatcgctatgccttcaagacggacccctcggtgttcgatactcgacaggcagcacagcctttacgccgggcgttcaagcggcctcgacgtgggatgtcaatttgatccgcgaacgtggacagttcatcggtgaggaggtgaaggcctcggggattcatgtcatacttggtcctgtggctgggccgctgggaaagactccgcagggcggtcgcaactgggagggcttcggtgtcgatccatatctcacgggcattgccatgggtcaaaccatcaacggcatccagtcggtaggcgtgcaggcgacagcgaagcactatatcctcaacgagcaggagctcaatcgagaaaccatttcgagcaacccagatgaccgaactctccatgagctgtatacttggccatttgccgacgcggttcaggccaatgtcgcttctgtcatgtgctcgtacaacaaggtcaataccacctgggcctgcgaggatcagtacacgctgcagactgtgctgaaagaccagctggggttcccaggctatgtcatgacggactggaacgcacagcacacgactgtccaaagcgcgaattctgggcttgacatgtcaatgcctggcacagacttcaacggtaacaatcggctctggggtccagctctcaccaatgcggtaaatagcaatcaggtccccacgagcagagtcgacgatatggtgactcgtatcctcgccgcatggtacttgacaggccaggaccaggcaggctatccgtcgttcaacatcagcagaaatgttcaaggaaaccacaagaccaatgtcagggcaattgccagggacggcatcgttctgctcaagaatgacgccaacatcctgccgctcaagaagcccgctagcattgccgtcgttggatctgccgcaatcattggtaaccacgccagaaactcgccctcgtgcaacgacaaaggctgcgacgacggggccttgggcatgggttggggttccggcgccgtcaactatccgtacttcgtcgcgccctacgatgccatcaataccagagcgtcttcgcagggcacccaggttaccttgagcaacaccgacaacacgtcctcaggcgcatctgcagcaagaggaaaggacgtcgccatcgtcttcatcaccgccgactcgggtgaaggctacatcaccgtggagggcaacgcgggcgatcgcaacaacctggatccgtggcacaacggcaatgccctggtccaggcggtggccggtgccaacagcaacgtcattgttgttgtccactccgttggcgccatcattctggagcagattcttgctcttccgcaggtcaaggccgttgtctgggcgggtcttccttctcaggagagcggcaatgcgctcgtcgacgtgctgtggggagatgtcagcccttctggcaagctggtgtacaccattgcgaagagccccaatgactataacactcgcatcgtttccggcggcagtgacagcttcagcgagggactgttcatcgactataagcacttcgacgacgccaatatcacgccgcggtacgagttcggctatggactgtcttacaccaagttcaactactcacgcctctccgtcttgtcgaccgccaagtctggtcctgcgactggggccgttgtgccgggaggcccgagtgatctgttccagaatgtcgcgacagtcaccgttgacatcgcaaactctggccaagtgactggtgccgaggtagcccagctgtacatcacctacccatcttcagcacccaggacccctccgaagcagctgcgaggctttgccaagctgaacctcacgcctggtcagagcggaacagcaacgttcaacatccgacgacgagatctcagctactgggacacggcttcgcagaaatgggtggtgccgtcggggtcgtttggcatcagcgtgggagcgagcagccgggatatcaggctgacgagcactctgtcggtagcg)( atggcgccctcagttacactgccgttgaccacggccatcctggccattgcccggctcgtcgccgcccagcaaccgggtaccagcacccccgaggtccatcccaagttgacaacctacaagtgtacaaagtccggggggtgcgtggcccaggacacctcggtggtccttgactggaactaccgctggatgcacgacgcaaactacaactcgtgcaccgtcaacggcggcgtcaacaccacgctctgccctgacgaggcgacctgtggcaagaactgcttcatcgagggcgtcgactacgccgcctcgggcgtcacgacctcgggcagcagcctcaccatgaaccagtacatgcccagcagctctggcggctacagcagcgtctctcctcggctgtatctcctggactctgacggtgagtacgtgatgctgaagctcaacggccaggagctgagcttcgacgtcgacctctctgctctgccgtgtggagagaacggctcgctctacctgtctcagatggacgagaacgggggcgccaaccagtataacacggccggtgccaactacgggagcggctactgcgatgctcagtgccccgtccagacatggaggaacggcaccctcaacactagccaccagggcttctgctgcaacgagatggatatcctggagggcaactcgagggcgaatgccttgacccctcactcttgcacggccacggcctgcgactctgccggttgcggcttcaacccctatggcagcggctacaaaagctactacggccccggagataccgttgacacctccaagaccttcaccatcatcacccagttcaacacggacaacggctcgccctcgggcaaccttgtgagcatcacccgcaagtaccagcaaaacggcgtcgacatccccagcgcccagcccggcggcgacaccatctcgtcctgcccgtccgcctcagcctacggcggcctcgccaccatgggcaaggccctgagcagcggcatggtgctcgtgttcagcatttggaacgacaacagccagtacatgaactggctcgacagcggcaacgccggcccctgcagcagcaccgagggcaacccatccaacatcctggccaacaaccccaacacgcacgtcgtcttctccaacatccgctggggagacattgggtctactacgaactcgactgcgcccccgcccccgcctgcgtccagcacgacgttttcgactacacggaggagctcgacgacttcgagcagcccgagctgcacgcagactcactgggggcagtgcggtggcattgggtacagcgggtgcaagacgtgcacgtcgggcactacgtgccagtatagcaacgactactactcgcaatgcctt)tctagaagcggcagcagcagcggcagcagcagcctggcgggcattgaaggccgcagcagcagcggcagcagcagcggcagcatgctgctgcccgtccccctgctgctaggcctgctgggcgccgccgccgacgtgaagcttatggcctcctccgagaacgtcatcaccgagttcatgcgcttcaaggtgcgcatggagggcaccgtgaacggccacgagttcgagatcgagggcgagggcgagggccgcccctacgagggccacaacaccgtgaagctgaaggtgaccaagggcggccccctgcccttcgcctgggacatcctgtccccccagttccagtacggctccaaggtgtacgtgaagcaccccgccgacatccccgactacaagaagctgtccttccccgagggcttcaagtgggagcgcgtgatgaacttcgaggacggcggcgtggcgaccgtgacccaggactcctccctgcaggacggctgcttcatctacaaggtgaagttcatcggcgtgaacttcccctccgacggccccgtgatgcagaagaagaccatgggctgggaggcctccaccgagcgcctgtacccccgcgacggcgtgctgaagggcgagacccacaaggccctgaagctgaaggacggcggccactacctggtggagttcaagtccatctacatggccaagaagcccgtgcagctgcccggctactactacgtggacgccaagctggacatcacctcccacaacgaggactacaccatcgtggagcagtacgagcgcaccgagggccgccaccacctgttcctgaaggacgagctgtaaagtagatgccgaccggatcgatccacttaacgttactgaaatcatcaaacagcttgacgaatctggatataagatcgttggtgtcgatgtcagctccggagttgagacaaatggtgttcaggatctcgataagatacgttcatttgtccaagcagcaaagagtgccttctagtgatttaatagctccatgtcaacaagaataaaacgcgttttcgggtttacctcttccagatacagctcatctgcaatgcattaatgcattgactgcaacctagtaacgccttcaggctccggcgaagagaagaatagcttagcagagctattttcattttcgggagacgagatcaagcagatcaacggtcgtcaagagacctacgagactgaggaatccgctcttggctccacgcgactatatatttgtctctaattgtactttgacatgctcctcttctttactctgatagcttgactatgaaaattccgtcaccagccctggcggccgccaccgcggtggagctccagcttttgttccctttagtgagggttaattccgagcttggcgtaatcatggtcatagctgtttcctgtgtgaaattgttatccgctcacaattccacacaacatacgagccggaagcataaagtgtaaagcctggggtgcctaatgagtgagctaactcacattaattgcgttgcgctcactgcccgctttccagtcgggaaacctgtcgtgccagctgcattaatgaatcggccaacgcgcggggagaggcggtttgcgtattgggcgctcttccgcttcctcgctcactgactcgctgcgctcggtcgttcggctgcggcgagcggtatcagctcactcaaaggcggtaatacggttatccacagaatcaggggataacgcaggaaagaacatgaaggctggcgtaatagcgaagaggcccgcaccgatcgcccttcccaacagttgcgcagcctgaatggcgaatgctagagcagcttgagcttggatcagattgtcgtttcccgccttcagtttaaactatcagtgtttgacaggatatattggcgg**gtaaacctaagagaaaagagcgttta**ttagaataacggatatttaaaagggcgtgaaaaggtttatccgttcgtccatttgtatgtgcatgccaaccacagggttcccctcgggatcaaagtactttgatccaacccctccgctgctatagtgcagtcggcttctgacgttcagtgcagccgtcttctgaaaacgacatgtcgcacaagtcctaagttacgcgacaggctgccgccctgcccttttcctggcgttttcttgtcgcgtgttttagtcgcataaagtagaatacttgcgactagaaccggagacattacgccatgaacaagagcgccgccgctggcctgctgggctatgcccgcgtcagcaccgacgaccaggacttgaccaaccaacgggccgaactgcacgcggccggctgcaccaagctgttttccgagaagatcaccggcaccaggcgcgaccgcccggagctggccaggatgcttgaccacctacgccctggcgacgttgtgacagtgaccaggctagaccgcctggcccgcagcacccgcgacctactggacattgccgagcgcatccaggaggccggcgcgggcctgcgtagcctggcagagccgtgggccgacaccaccacgccggccggccgcatggtgttgaccgtgttcgccggcattgccgagttcgagcgttccctaatcatcgaccgcacccggagcgggcgcgaggccgccaaggcccgaggcgtgaagtttggcccccgccctaccctcaccccggcacagatcgcgcacgcccgcgagctgatcgaccaggaaggccgcaccgtgaaagaggcggctgcactgcttggcgtgcatcgctcgaccctgtaccgcgcacttgagcgcagcgaggaagtgacgcccaccgaggccaggcggcgcggtgccttccgtgaggacgcattgaccgaggccgacgccctggcggccgccgagaatgaacgccaagaggaacaagcatgaaaccgcaccaggacggccaggacgaaccgtttttcattaccgaagagatcgaggcggagatgatcgcggccgggtacgtgttcgagccgcccgcgcacgtctcaaccgtgcggctgcatgaaatcctggccggtttgtctgatgccaagctggcggcctggccggccagcttggccgctgaagaaaccgagcgccgccgtctaaaaaggtgatgtgtatttgagtaaaacagcttgcgtcatgcggtcgctgcgtatatgatgcgatgagtaaataaacaaatacgcaaggggaacgcatgaaggttatcgctgtacttaaccagaaaggcgggtcaggcaagacgaccatcgcaacccatctagcccgcgccctgcaactcgccggggccgatgttctgttagtcgattccgatccccagggcagtgcccgcgattgggcggccgtgcgggaagatcaaccgctaaccgttgtcggcatcgaccgcccgacgattgaccgcgacgtgaaggccatcggccggcgcgacttcgtagtgatcgacggagcgccccaggcggcggacttggctgtgtccgcgatcaaggcagccgacttcgtgctgattccggtgcagccaagcccttacgacatatgggccaccgccgacctggtggagctggttaagcagcgcattgaggtcacggatggaaggctacaagcggcctttgtcgtgtcgcgggcgatcaaaggcacgcgcatcggcggtgaggttgccgaggcgctggccgggtacgagctgcccattcttgagtcccgtatcacgcagcgcgtgagctacccaggcactgccgccgccggcacaaccgttcttgaatcagaacccgagggcgacgctgcccgcgaggtccaggcgctggccgctgaaattaaatcaaaactcatttgagttaatgaggtaaagagaaaatgagcaaaagcacaaacacgctaagtgccggccgtccgagcgcacgcagcagcaaggctgcaacgttggccagcctggcagacacgccagccatgaagcgggtcaactttcagttgccggcggaggatcacaccaagctgaagatgtacgcggtacgccaaggcaagaccattaccgagctgctatctgaatacatcgcgcagctaccagagtaaatgagcaaatgaataaatgagtagatgaattttagcggctaaaggaggcggcatggaaaatcaagaacaaccaggcaccgacgccgtggaatgccccatgtgtggaggaacgggcggttggccaggcgtaagcggctgggttgtctgccggccctgcaatggcactggaacccccaagcccgaggaatcggcgtgacggtcgcaaaccatccggcccggtacaaatcggcgcggcgctgggtgatgacctggtggagaagttgaaggccgcgcaggccgcccagcggcaacgcatcgaggcagaagcacgccccggtgaatcgtggcaagcggccgctgatcgaatccgcaaagaatcccggcaaccgccggcagccggtgcgccgtcgattaggaagccgcccaagggcgacgagcaaccagattttttcgttccgatgctctatgacgtgggcacccgcgatagtcgcagcatcatggacgtggccgttttccgtctgtcgaagcgtgaccgacgagctggcgaggtgatccgctacgagcttccagacgggcacgtagaggtttccgcagggccggccggcatggccagtgtgtgggattacgacctggtactgatggcggtttcccatctaaccgaatccatgaaccgataccgggaagggaagggagacaagcccggccgcgtgttccgtccacacgttgcggacgtactcaagttctgccggcgagccgatggcggaaagcagaaagacgacctggtagaaacctgcattcggttaaacaccacgcacgttgccatgcagcgtacgaagaaggccaagaacggccgcctggtgacggtatccgagggtgaagccttgattagccgctacaagatcgtaaagagcgaaaccgggcggccggagtacatcgagatcgagctagctgattggatgtaccgcgagatcacagaaggcaagaacccggacgtgctgacggttcaccccgattactttttgatcgatcccggcatcggccgttttctctaccgcctggcacgccgcgccgcaggcaaggcagaagccagatggttgttcaagacgatctacgaacgcagtggcagcgccggagagttcaagaagttctgtttcaccgtgcgcaagctgatcgggtcaaatgacctgccggagtacgatttgaaggaggaggcggggcaggctggcccgatcctagtcatgcgctaccgcaacctgatcgagggcgaagcatccgccggttcctaatgtacggagcagatgctagggcaaattgccctagcaggggaaaaaggtcgaaaaggtctctttcctgtggatagcacgtacattgggaacccaaagccgtacattgggaaccggaacccgtacattgggaacccaaagccgtacattgggaaccggtcacacatgtaagtgactgatataaaagagaaaaaaggcgatttttccgcctaaaactctttaaaacttattaaaactcttaaaacccgcctggcctgtgcataactgtctggccagcgcacagccgaagagctgcaaaaagcgcctacccttcggtcgctgcgctccctacgccccgccgcttcgcgtcggcctatcgcggccgctggccgctcaaaaatggctggcctacggccaggcaatctaccagggcgcggacaagccgcgccgtcgccactcgaccgccggcgcccacatcaaggcaccctgcctcgcgcgtttcggtgatgacggtgaaaacctctgacacatgcagctcccggagacggtcacagcttgtctgtaagcggatgccgggagcagacaagcccgtcagggcgcgtcagcgggtgttggcgggtgtcggggcgcagccatgacccagtcacgtagcgatagcggagtgtatactggcttaactatgcggcatcagagcagattgtactgagagtgcaccatatgcggtgtgaaataccgcacagatgcgtaaggagaaaataccgcatcaggcgctcttccgcttcctcgctcactgactcgctgcgctcggtcgttcggctgcggcgagcggtatcagctcactcaaaggcggtaatacggttatccacagaatcaggggataacgcaggaaagaacatgtgagcaaaaggccagcaaaaggccaggaaccgtaaaaaggccgcgttgctggcgtttttccataggctccgcccccctgacgagcatcacaaaaatcgacgctcaagtcagaggtggcgaaacccgacaggactataaagataccaggcgtttccccctggaagctccctcgtgcgctctcctgttccgaccctgccgcttaccggatacctgtccgcctttctcccttcgggaagcgtggcgctttctcatagctcacgctgtaggtatctcagttcggtgtaggtcgttcgctccaagctgggctgtgtgcacgaaccccccgttcagcccgaccgctgcgccttatccggtaactatcgtcttgagtccaacccggtaagacacgacttatcgccactggcagcagccactggtaacaggattagcagagcgaggtatgtaggcggtgctacagagttcttgaagtggtggcctaactacggctacactagaaggacagtatttggtatctgcgctctgctgaagccagttaccttcggaaaaagagttggtagctcttgatccggcaaacaaaccaccgctggtagcggtggtttttttgtttgcaagcagcagattacgcgcagaaaaaaaggatctcaagaagatcctttgatcttttctacggggtctgacgctcagtggaacgaaaactcacgttaagggattttggtcatgcattctaggtactaaaacaattcatccagtaaaatataatattttattttctcccaatcaggcttgatccccagtaagtcaaaaaatagctcgacatactgttcttccccgatatcctccctgatcgaccggacgcagaaggcaatgtcataccacttgtccgccctgccgcttctcccaagatcaataaagccacttactttgccatctttcacaaagatgttgctgtctcccaggtcgccgtgggaaaagacaagttcctcttcgggcttttccgtctttaaaaaatcatacagctcgcgcggatctttaaatggagtgtcttcttcccagttttcgcaatccacatcggccagatcgttattcagtaagtaatccaattcggctaagcggctgtctaagctattcgtatagggacaatccgatatgtcgatggagtgaaagagcctgatgcactccgcatacagctcgataatcttttcagggctttgttcatcttcatactcttccgagcaaaggacgccatcggcctcactcatgagcagattgctccagccatcatgccgttcaaagtgcaggacctttggaacaggcagctttccttccagccatagcatcatgtccttttcccgttccacatcataggtggtccctttataccggctgtccgtcatttttaaatataggttttcattttctcccaccagcttatataccttagcaggagacattccttccgtatcttttacgcagcggtatttttcgatcagttttttcaattccggtgatattctcattttagccatttattatttccttcctcttttctacagtatttaaagataccccaagaagctaattataacaagacgaactccaattcactgttccttgcattctaaaaccttaaataccagaaaacagctttttcaaagttgttttcaaagttggcgtataacatagtatcgacggagccgattttgaaaccgcggtgatcacaggcagcaacgctctgtcatcgttacaatcaacatgctaccctccgcgagatcatccgtgtttcaaacccggcagcttagttgccgttcttccgaatagcatcggtaacatgagcaaagtctgccgccttacaacggctctcccgctgacgccgtcccggactgatgggctgcctgtatcgagtggtgattttgtgccgagctgccggtcggggagctgttggctggctggtggcaggatatattgtggtgtaaacaaattgacgcttagacaacttaataacacattgcggacgtttttaatgtactgaattaacgccgaattaattcgggggatctggattttagtactggattttggttttaggaattagaaattttattgatagaagtattttacaaatacaaatacatactaagggtttcttatatgctcaacacatgagcgaaaccctataggaaccctaattcccttatctgggaactactcacacattattatggagaaacttggcaa

Fig. S1 Sequences of plasmid Px-DsRed. Pcbh, Ttrpc, hyg, linker, and XbaⅠwere represented in light green, pink, dark green, blue and light red respectively; CEL3A, CEL7A, CEL7B and DsRed were marked in purple, yellow, dark red and grey, respectively; The LB and RB of the binary vector were underlined and in bold.


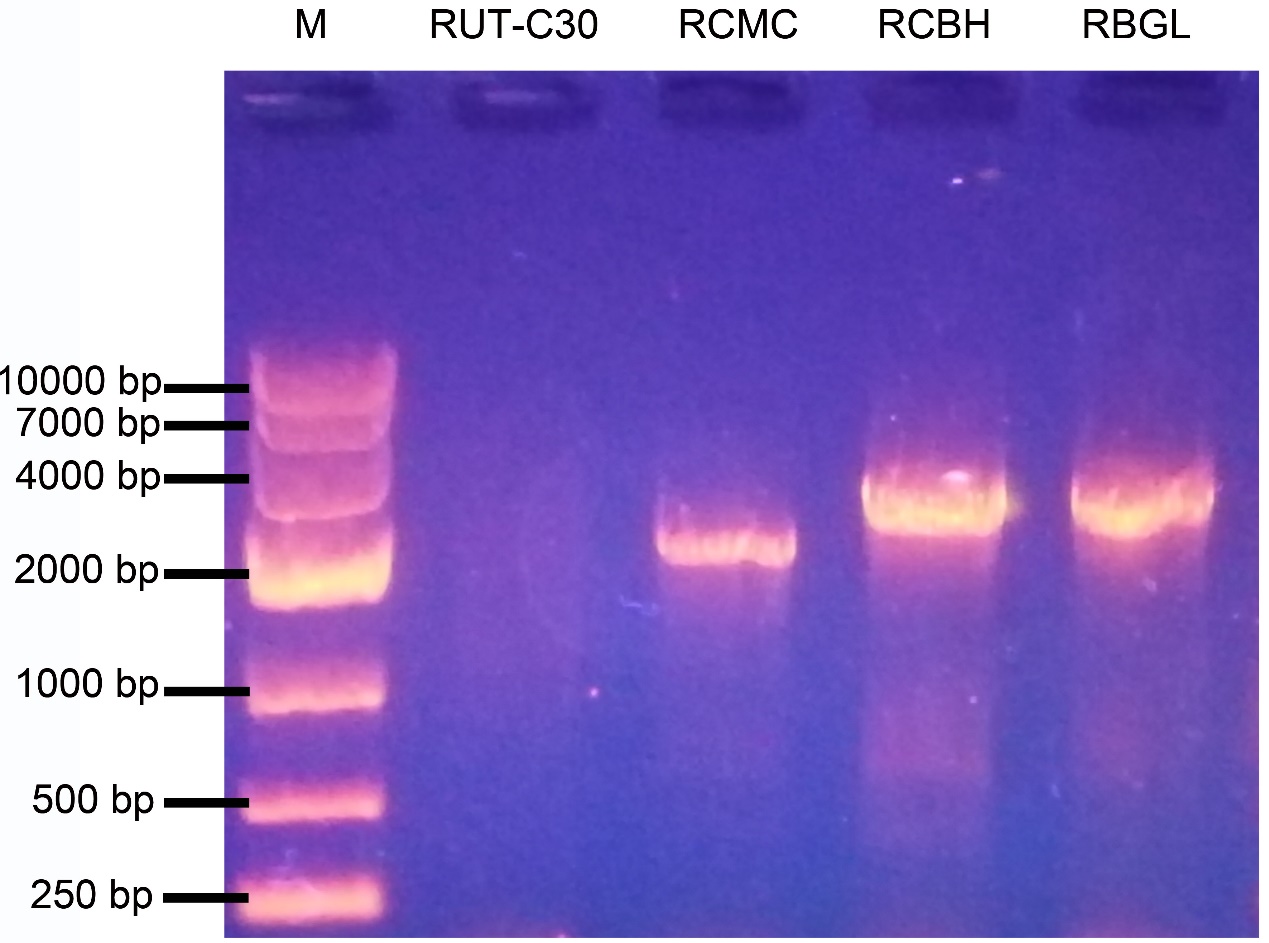


Fig. S2 PCR conformation results for RUT-C30 and the transformants RCMC, RCBH and RBG.

**
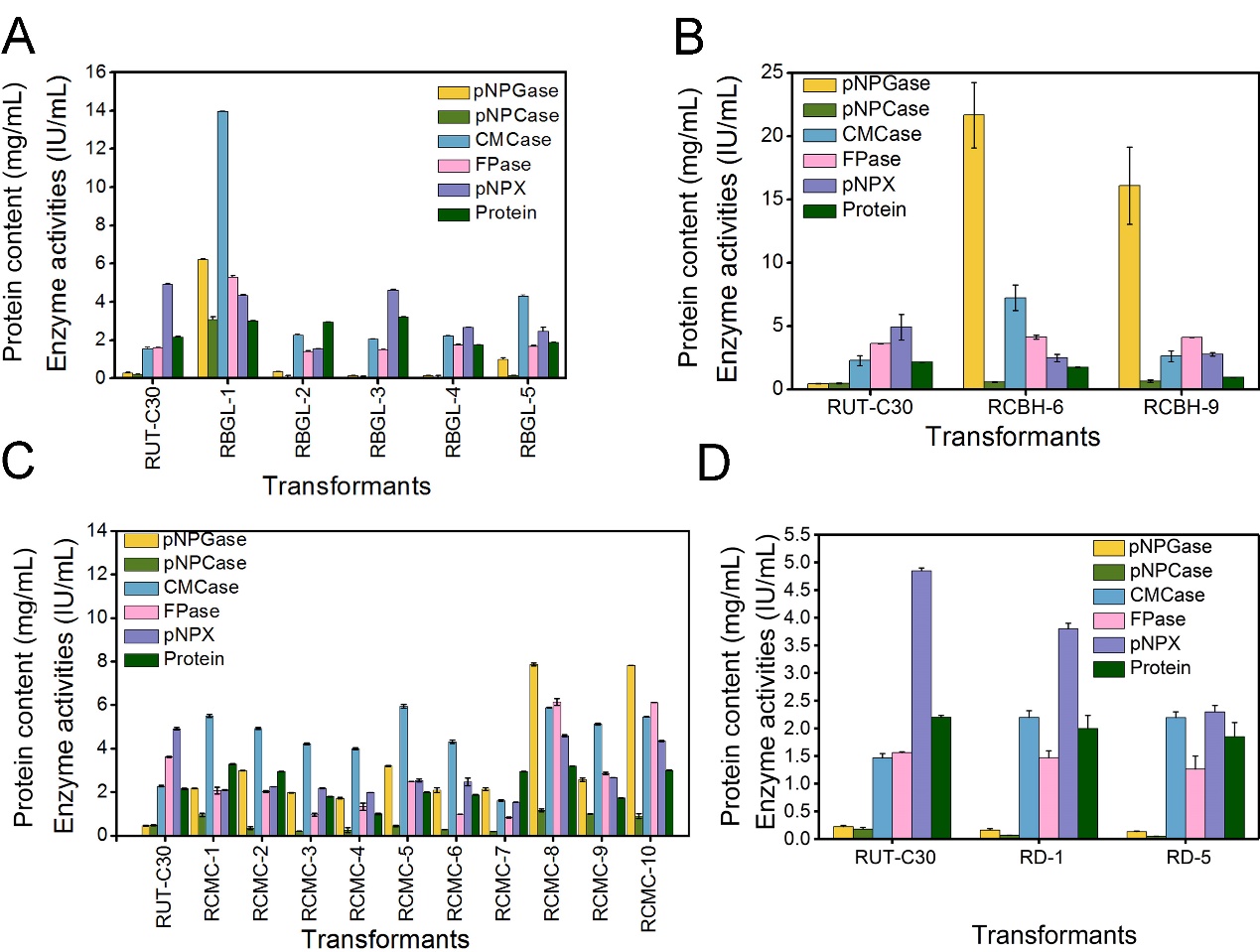
**

Fig. S3 Cellulase activities of recombinant *T. reesei* strains RBGL (A), RCBH (B), RCMC (C), and RD (D). The mutants were grown in TMM+2% cellulose for 5 days. pNPGase: the β-glucosidase activity; pNPCase: the CBH activity; CMCase: the CMC activity; FPase: the filter paper activity. Data are represented as the mean of three independent experiments and error bars expressed the standard deviations.


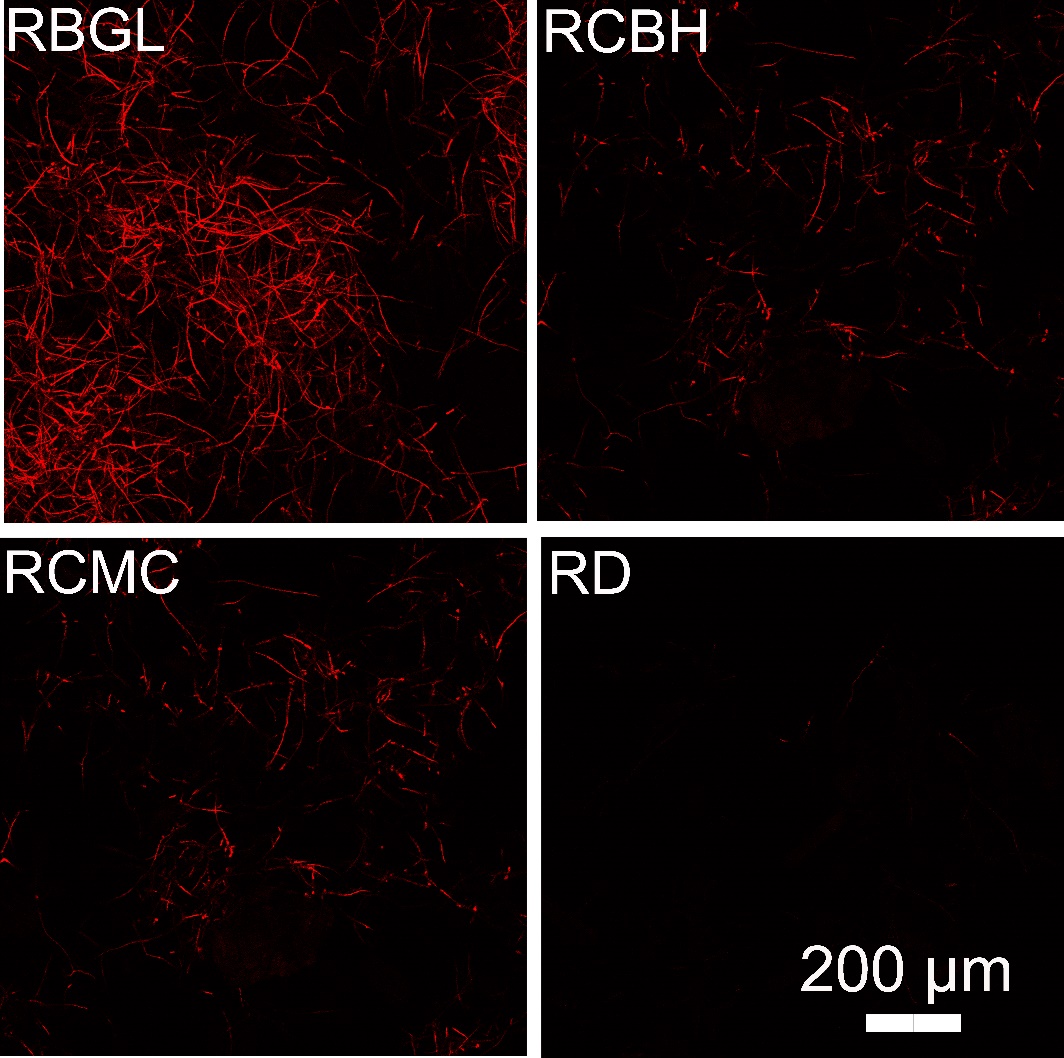


Fig. S4 CLSM imaging of RBGL, RCBH, RCMC, and RD cultivated in TMM+2% cellulose for 5 days using 10 x objective.


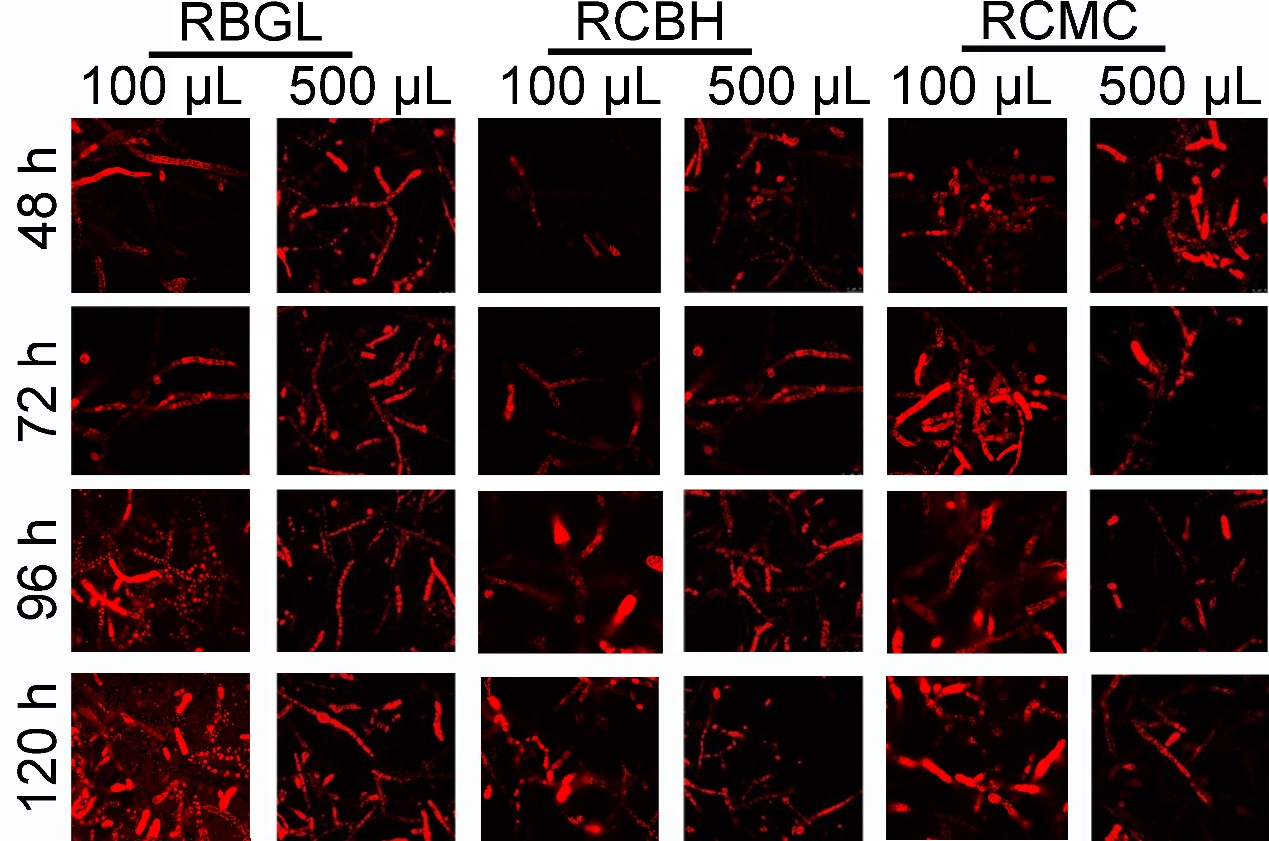


Fig. S5 Confocal images of recombinant strains RBGL, RCBH and RCMC cultured in TMM + 2% cellulose + 100 μL or 500 μL DMSO for 48, 72, 96 and 120 h.


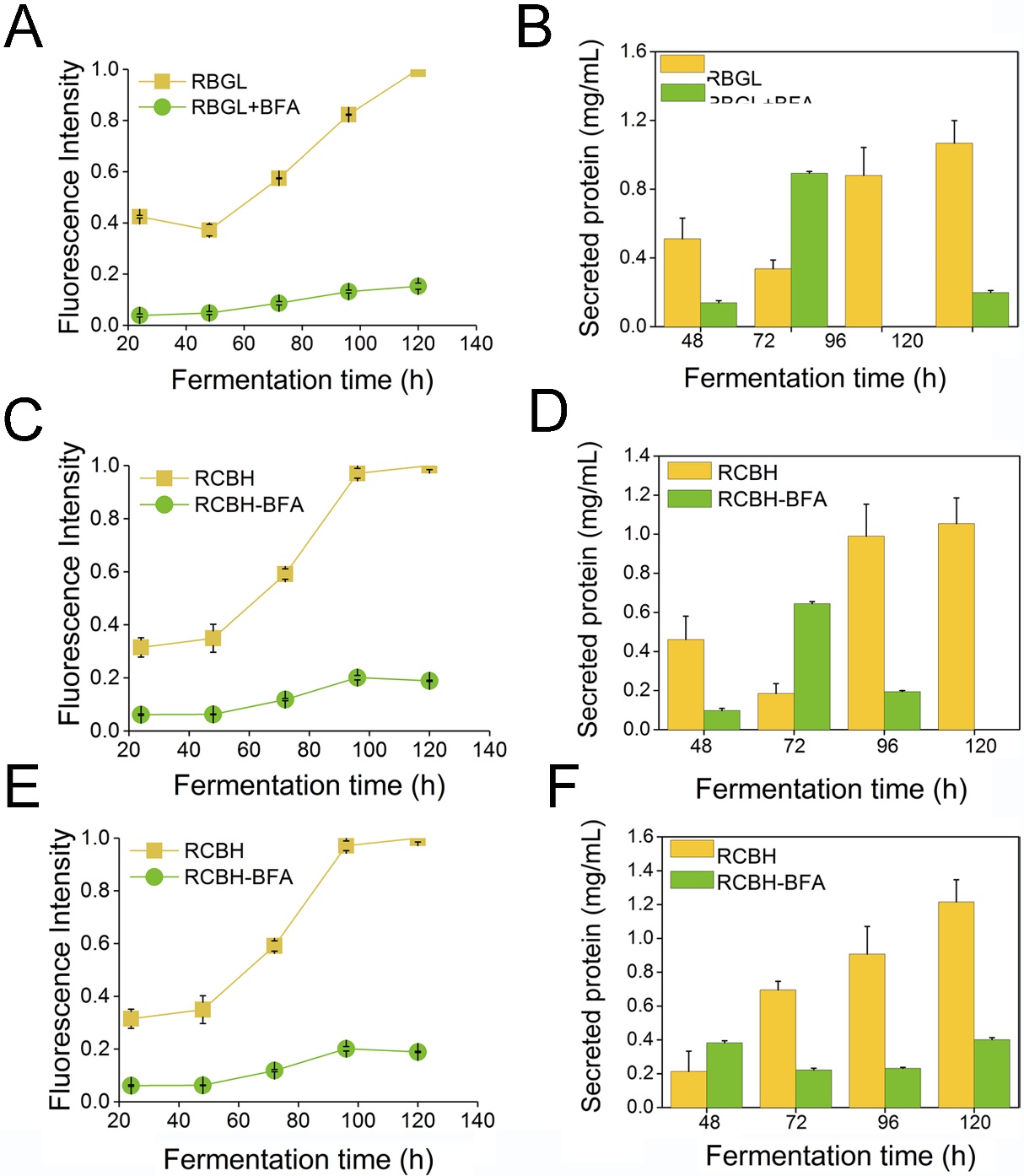


Fig. S6 Fluorescence intensity (A, C, and E), and secreted protein concentration (B, D, and F) in the culture supernatants of recombinant strains RBGL, RCBH and RCMC cultured in TMM+2% cellulose + 50 μg/mL BFA for 48, 72, 96 and 120 h.

Table S1 PCR primers for plasmid construction

| Primer | Sequence (5’to 3’) |
| --- | --- |
| cel7a-dsred Fw | ACCCAATAGTCAATCTAGAATGTATCGGAAGTTGGCCGT |
| cel7a-dsred Rv | CTGCTGCTGCCGCTTCTAGACAGGCACTGAGAGTAGTAAGGGTTG |
| cel7b-dsred Fw | ACCCAATAGTCAATCTAGAATGGCGCCCTCAGTTACACTGC |
| cel7b-dsred Rv | CTGCTGCTGCCGCTTCTAGAAAGGCATTGCGAGTAGTAGTCGTTGCTATACTGGCACG |
| cel3a-dsred Fw | ACCCAATAGTCAATCTAGAATGCGTTACCGAACAGCAGCTG |
| cel3a-dsred Rv | CTGCTGCTGCCGCTTCTAGACGCTACCGACAGAGTGCTCGTCA |

Table S2 PCR primers for confirmation of transformants

| Primer | Sequence (5’to 3’) |
| --- | --- |
| pDHT -RCBH-yz -F | CTCCGGGCAAATGCAAAGTGTGG |
| pDHT -RCBH-yz -R | GTCACCTTCAGCTTCACGGT |
| pDHT -RCMC-yz -F | CTCCGGGCAAATGCAAAGTGTGG |
| pDHT -RCMC-yz -R | GTCACCTTCAGCTTCACGGT |
| pDHT -RBGL-yz -F | CTCCGGGCAAATGCAAAGTGTGG |
| pDHT -RBGL-yz -R | GTCACCTTCAGCTTCACGGT |
